# Shark nanobodies with potent SARS-CoV-2 neutralizing activity and broad sarbecovirus reactivity

**DOI:** 10.1038/s41467-023-36106-x

**Published:** 2023-02-03

**Authors:** Wei-Hung Chen, Agnes Hajduczki, Elizabeth J. Martinez, Hongjun Bai, Hanover Matz, Thomas M. Hill, Eric Lewitus, William C. Chang, Layla Dawit, Caroline E. Peterson, Phyllis A. Rees, Adelola B. Ajayi, Emily S. Golub, Isabella Swafford, Vincent Dussupt, Sapna David, Sandra V. Mayer, Sandrine Soman, Caitlin Kuklis, Courtney Corbitt, Jocelyn King, Misook Choe, Rajeshwer S. Sankhala, Paul V. Thomas, Michelle Zemil, Lindsay Wieczorek, Tricia Hart, Debora Duso, Larry Kummer, Lianying Yan, Spencer L. Sterling, Eric D. Laing, Christopher C. Broder, Jazmean K. Williams, Edgar Davidson, Benjamin J. Doranz, Shelly J. Krebs, Victoria R. Polonis, Dominic Paquin-Proulx, Morgane Rolland, William W. Reiley, Gregory D. Gromowski, Kayvon Modjarrad, Helen Dooley, M. Gordon Joyce

**Affiliations:** 1 Emerging Infectious Diseases Branch, Walter Reed Army Institute of Research, Silver Spring, MD USA; 2 Henry M. Jackson Foundation for the Advancement of Military Medicine, Bethesda, MD USA; 3 U.S. Military HIV Research Program, Walter Reed Army Institute of Research, Silver Spring, MD USA; 4 Department of Microbiology and Immunology, University of Maryland School of Medicine, Baltimore, MD USA; 5 Institute of Marine and Environmental Technology, Baltimore, MD USA; 6 Viral Diseases Branch, Walter Reed Army Institute of Research, Silver Spring, MD USA; 7 Trudeau Institute, Saranac Lake, NY USA; 8 Department of Microbiology and Immunology, Uniformed Services University, Bethesda, MD USA; 9 Integral Molecular, Philadelphia, PA USA

**Keywords:** Viral infection, X-ray crystallography, Applied immunology, Antibody therapy

## Abstract

Despite rapid and ongoing vaccine and therapeutic development, SARS-CoV-2 continues to evolve and evade, presenting a need for next-generation diverse therapeutic modalities. Here we show that nurse sharks immunized with SARS-CoV-2 recombinant receptor binding domain (RBD), RBD-ferritin (RFN), or spike protein ferritin nanoparticle (SpFN) immunogens elicit a set of new antigen receptor antibody (IgNAR) molecules that target two non-overlapping conserved epitopes on the spike RBD. Representative shark antibody variable NAR-Fc chimeras (ShAbs) targeting either of the two epitopes mediate cell-effector functions, with high affinity to all SARS-CoV-2 viral variants of concern, including the divergent Omicron strains. The ShAbs potently cross-neutralize SARS-CoV-2 WA-1, Alpha, Beta, Delta, Omicron BA.1 and BA.5, and SARS-CoV-1 pseudoviruses, and confer protection against SARS-CoV-2 challenge in the K18-hACE2 transgenic mouse model. Structural definition of the RBD-ShAb01-ShAb02 complex enabled design and production of multi-specific nanobodies with enhanced neutralization capacity, and picomolar affinity to divergent sarbecovirus clade 1a, 1b and 2 RBD molecules. These shark nanobodies represent potent immunotherapeutics both for current use, and future sarbecovirus pandemic preparation.

## Abstract (web summary)

SARS-CoV-2 variants of concern continue to emerge, reducing vaccine efficacy and limiting therapeutic options. Here, Chen and colleagues describe the identification and design of shark nanobodies with pansarbecovirus activity.

## Introduction

Severe Acute Respiratory Syndrome Coronavirus-2 (SARS-CoV-2), the causative agent of the Coronavirus Disease-19 (COVID-19) pandemic, belongs to a genus of coronaviruses (CoVs) that causes significant disease in humans^1,2^. CoVs have the propensity to shift between zoonotic reservoirs and into human populations, making them a continual global health threat. Of the CoVs that are known to infect humans, the betacoronaviruses (notable examples include MERS-CoV, SARS-CoV-1, and SARS-CoV-2) cause the highest mortality^3,4^. However, additional alpha-CoVs cause illness in humans, and hundreds more CoVs circulate in animal populations with the potential for zoonotic spillover^5–7^. Despite the successfully rapid development of safe and effective SARS-CoV-2 vaccines, there remains an urgent need for next-generation prophylactics, therapeutics and diagnostic reagents. The continual emergence of divergent SARS-CoV-2 Variants of Concern (VoC) with increased transmissibility, viral sequence divergence and potential for immune escape, further underscores the specific need for broadly effective monoclonal antibodies^8^.

Camelids and cartilaginous fishes, including sharks, have antibodies that lack light chains^9^. Binding domains derived from these antibodies, so-called nanobodies, are of specific interest due to their small size and robust stability^10–12^. The cartilaginous fish heavy-chain antibody isotype, new antigen receptor (IgNAR), was first described in the nurse shark (*Ginglymostoma cirratum*)^13^. The variable binding domains of these antibodies, VNARs, are the smallest known natural nanobodies. Despite their small size, VNARs are capable of generating a wide paratope repertoire due to their complementarity-determining region 3 (CDR3) recombination process where two or more D segments are combined^14^. VNAR domains are most closely related to cartilaginous fish TCR domains^15^ with a β-sandwich fold containing eight β-strands, and shortened framework region 2 (FR2) and CDR2 loops. While having only two true CDRs, high-rates of somatic mutation are observed in two additional hypervariable loops (HV2 and HV4), which have been shown to contribute to high-affinity antigen binding^16^.

In this work, we identify unique molecules with affinity for SARS-CoV-2 and other sarbecoviruses, by immunizing nurse sharks with SARS-CoV-2 spike receptor binding domain (RBD), RBD-ferritin (RFN), and spike-ferritin nanoparticles (SpFN), all based on the original SARS-CoV-2 Wuhan-1 virus sequence^17–19^. This set of VNARs have broad binding activity, neutralization capacity and protective efficacy, and we structurally characterize the recognition properties of these nanobodies. Using this structural information, we design a set of multi-specific antibodies with increased neutralization breadth and potency.

## Results

### Shark immunization yields high-affinity anti-SARS-CoV-2 nanobodies

**Fig. 1:**
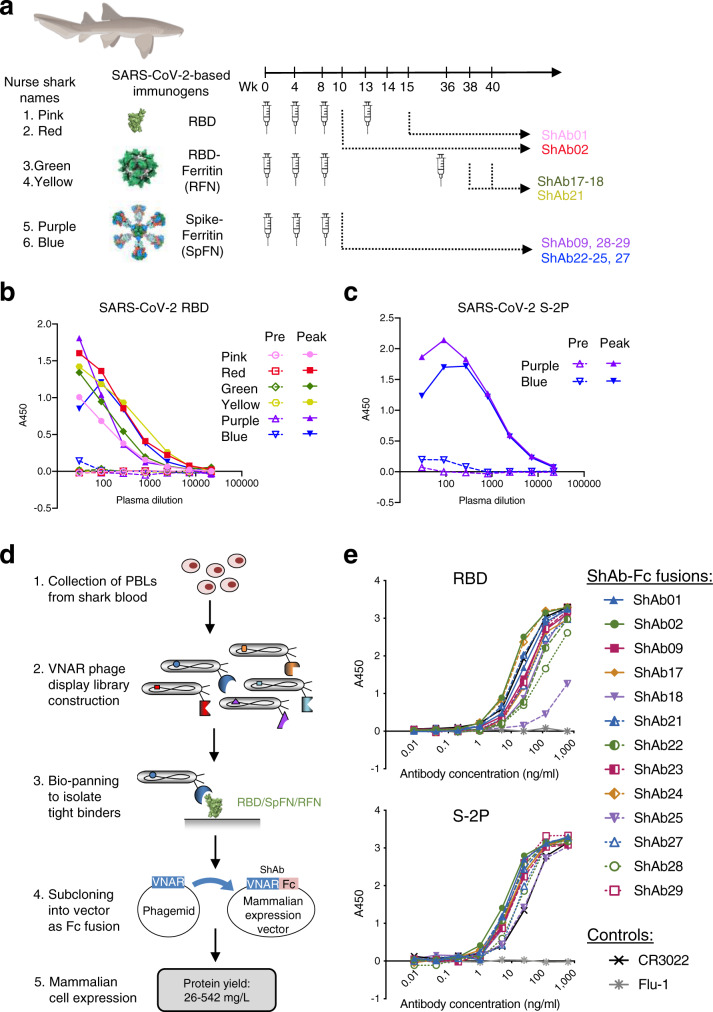
Induction of shark IgNAR antibody responses and isolation of antigen-specific VNARs. **a** Immunization schedule of six nurse sharks for VNAR isolation. Sharks “Pink” and “Red” were immunized with SARS-CoV-2 RBD protein, followed by panning and identification of ShAb01 and ShAb02. Nurse sharks ‘Green’ and ‘Yellow’ were immunized with SARS-CoV-2 RBD-ferritin nanoparticle, followed by panning and identification of ShAb17-18 & 21 VNAR molecules. Nurse sharks ‘Purple’ and ‘Blue’ were immunized with SARS-CoV-2 Spike-ferritin nanoparticle, followed by panning and identification of ShAb09 and ShAb22-25 & 27–29 VNAR molecules. **b** Shark IgNAR peak plasma titers from all animals tested against SARS-CoV-2 RBD by ELISA. Pre-bleed samples serve as the negative control for each animal. **c** Shark IgNAR peak plasma titers from SpFN immunized animals (‘Purple’ and ‘Blue’) tested against SARS-CoV-2 S-2P by ELISA. Pre-bleed samples serve as the negative control for each animal. **d** Flow-chart depicting the steps involved from initial shark peripheral blood lymphocyte (PBL) isolation to ShAb protein expression. **e** ELISA of ShAb-Fc molecules against SARS-CoV-2 RBD (top) and S-2P trimer (bottom). Background binding to blocking agent, bovine serum albumin, has been subtracted from each data point. Source data are provided as a Source Data file. The shark image was created with BioRender.com.

Sharks were immunized with three different immunogens (Fig. 1a, Supplementary Table 1). The first pair of sharks designated ‘Pink’ and ‘Red’, were immunized with SARS-CoV-2 RBD. Sharks ‘Green’ and ‘Yellow’ were immunized with the RFN immunogen^19^. The third group of sharks ‘Purple’ and ‘Blue’, were immunized with the SpFN immunogen^17,18,20^. Immunogens were adjuvanted with complete Freund’s adjuvant for the prime, and incomplete Freund’s adjuvant for the second immunization; subsequent immunizations were unadjuvanted and administered intravenously. Shark blood samples were collected from the caudal vein, two weeks after each immunization and responses assayed by an IgNAR-specific ELISA against SARS-CoV-2 RBD and a stabilized trimeric S protein (S-2P)^21^. The peak responses for each animal are shown in Fig. 1b, c. To capture the repertoire of VNARs elicited by the immunizations, a phage-display library was generated for each of the six sharks (Fig. 1d). These VNAR libraries were screened using different panning strategies (Supplementary Table 1); libraries from the RBD-immunized animals were panned on RBD, while the remaining libraries were panned on SpFN or RFN. Unique antigen-positive VNARs were expressed as human IgG1 Fc-fusion chimeras (ShAbs) by subcloning the phagemid VNAR insert sequence into a mammalian expression vector upstream of the human IgG1 Fc encoding sequence (Fig. 1d, Supplementary Fig. 1). These fusion proteins, from here on referred to as ShAb01, etc., showed high protein expression levels via transient transfection of Expi293F mammalian cells with purified yields ranging from 26 to 542 mg/L (Supplementary Fig. 1). The ShAb molecules were purified using Protein A affinity and assayed by ELISA for binding to SARS-CoV-2 RBD and S-2P (Fig. 1e). All ShAb molecules bound to both target proteins.

### Elicited nanobodies fall within two distinct antigenic groups based on their RBD-binding profile

**Fig. 2:**
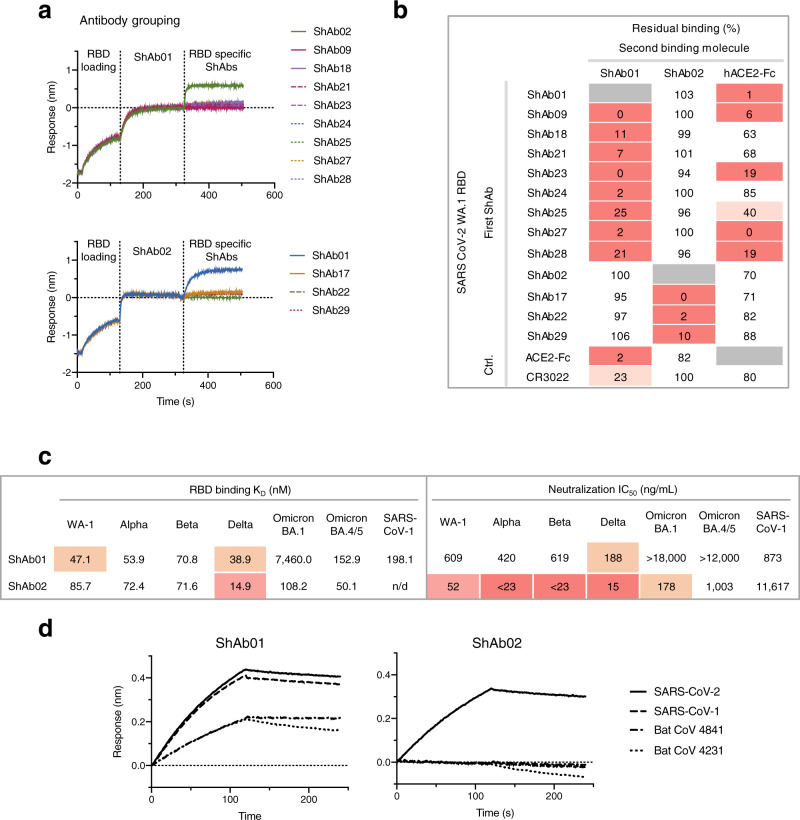
Antigenic characterization of the ShAb molecules. **a** ShAb RBD-competition assay assessed by Bio-Layer Interferometry (BLI). BLI measurements are performed with immobilized SARS-CoV-2 RBD and ShAb in solution. ShAb01 (top) or ShAb02 (bottom) are bound to the RBD molecule followed by incubation with other ShAbs. **b** Epitope binning of the ShAb molecules. Values represent the % residual binding of the indicated second molecule (ShAb01, ShAb02, ACE2, or CR3022) after saturation of the antigen (WA-1 RBD) with the indicated first antibody (left column). Shading from dark to light indicates competition strength ranging from strong competition (0–33%), to reduced competition (> 50%). Competition groups are indicated by black boxes. Human ACE2-Fc and CR3022 were used as controls. **c** RBD kinetic binding constants, and neutralization IC_50_ titers of SARS-CoV-2 viral VoC and SARS-CoV-1 pseudoviruses by ShAb01 and ShAb02. n/d indicates no binding was detected. **d** ShAb01 and ShAb02 binding assays performed by BLI with immobilized ShAbs and SARS-CoV-1 RBD and SARS-CoV-2 RBD, or clade 1b bat sarbecovirus trimeric S molecules in solution. Source data are provided as a Source Data file.

Initially, two Fc-chimera molecules, ShAb01 and ShAb02, isolated from sharks ‘Pink’ and ‘Red’, were produced and characterized prior to the identification of additional RBD-targeting VNARs from the additional four sharks immunized with RFN or SpFN (Fig. 1a). Epitope competition studies using biolayer interferometry showed that these additional VNAR-Fc chimeric ShAb antibodies blocked either ShAb01 or ShAb02 binding (Fig. 2a, b), forming two distinct antigenic groups with respect to their RBD binding properties. The ShAb01 antigenic group showed the greatest capacity to block ACE2 binding, as well as the class-IV antibody CR3022 (Fig. 2b). ShAb01 and ShAb02 were chosen as representatives from the two antigenic groups for further assessment. Both ShAb01 and ShAb02 show robust binding to both SARS-CoV-2 RBD and S-2P molecules, with affinity in the nanomolar range (Fig. 2c, Supplementary Fig. 2, Supplementary Table 2). K_D_ values for both ShAbs were in the low nanomolar range (14.9–85.7 nM) against SARS-CoV-2 RBD WA-1 and VOC including Alpha, Beta, Gamma, and Delta. ShAb01 had low affinity to the Omicron BA.1, BA.2 and BA.2.12.1 VoC, while ShAb02 maintained high affinity to the Omicron variants tested with K_D_ values at 50–125 nM (Supplementary Table 2). Furthermore, ShAb01 showed cross-reactivity to SARS CoV-1 RBD and two bat sarbecovirus S molecules as assessed by biolayer interferometry (Fig. 2d).

ShAb01 and ShAb02 were also assessed for their ability to neutralize SARS-CoV-1 and a panel of SARS-CoV-2 pseudoviruses (Fig. 2c). ShAb01 displayed potent neutralization against SARS-CoV-2 WA-1, Alpha, Beta and Delta variants and the heterologous SARS-CoV-1 Urbani strain with IC_50_ values of 188–873 ng/ml. ShAb02 neutralized SARS-CoV-2 WA-1, Alpha, Beta, and Delta variants, with IC_50_ values of 15–52 ng/ml, while having reduced neutralization potency against SARS-CoV-1 with an IC_50_ of 11.6 μg/ml (Fig. 2c). Neutralization of the Omicron BA.1 variant matched closely to the RBD binding data, with minimal neutralization observed for ShAb01, while ShAb02 maintained neutralization against the Omicron BA.1 and BA.4/5 variants with neutralization IC_50_ value of 178 ng/ml and 1 ug/ml, respectively.

### ShAbs protect against lethal virus challenge in the K18-hACE2 mouse model

**Fig. 3:**
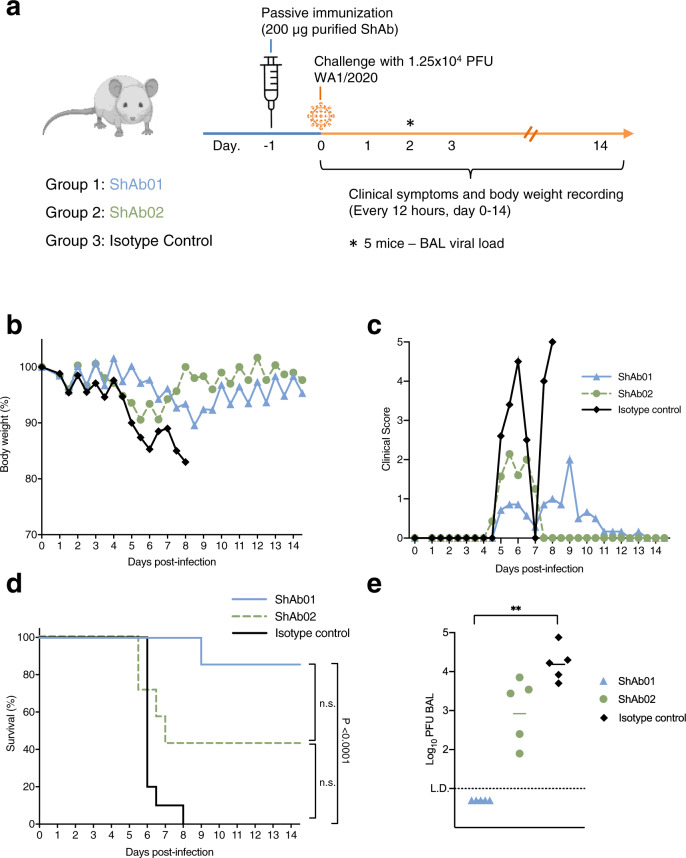
Passive immunization with ShAb01 and ShAb02 protect mice from SARS-CoV-2 challenge. **a** Schematic of K18-hACE2 mice SARS-CoV-2 challenge study. Mice (*n* = 13/group, 7 female, 6 male; 15/group for the isotype control, 8 female, 7 male) received an intraperitoneal injection of ShAb01 (blue), or ShAb02 (green), or IgG1 isotype control mAb (black), one day prior to challenge with 1.25 × 10^4^ PFU of SARS-CoV-2 virus (WA-1/2020). A cohort of mice (*n* = 5/group) were sacrificed 2 days post-challenge with the BAL fluid analyzed for viral load. The remaining mice (*n* = 8/group or 10 for the isotype control group) were assessed daily for weight and clinical symptoms. **b** Body weight measurements for K18-hACE2 mice. Percentage of initial weight is plotted. Isotype control mAb (black diamond), or ShAb01 (blue triangle) or ShAb02 (green circle). **c** Clinical score measurements of the K18-hACE2 study groups. All animals in the isotype control group were euthanized by study day 8. **d** Survival of K18-hACE2 mice for the 3 study groups. Key is shown in panel. Survival curves were compared individually to the isotype control using a Mantel-Cox log-rank test, and the significant *P*-value (*P* < 0.0001) is shown. n.s. indicates not significant differences. **e** SARS-CoV-2 viral loads in BAL were measured 2 days post-challenge in a subset of animals (*n* = 5/group) by plaque assay. Asterisks indicate significance compared to the antibody isotype control group by one-way ANOVA with Dunn’s multiple comparisons test, **P < 0.01. Source data are provided as a Source Data file. The mouse image was created with BioRender.com.

To assess the ability of the ShAb molecules to protect against SARS-CoV-2 infection, we carried out an in vivo protection study using K18-hACE2 transgenic mice, which express human ACE2 on their epithelial cells, making them susceptible to SARS-CoV-2 infection (Fig. 3). Groups of 13 mice were passively immunized by intraperitoneal injection of 200 μg (equivalent to 10 mg per kg body weight) of ShAb01, ShAb02, or an IgG1 isotype control, followed 24 h later by intranasal challenge with 1.25 × 10^4^ PFU of SARS-CoV-2 WA-1/2020. The mice were monitored for 14 days for weight loss, survival, and clinical score (Fig. 3a). Both ShAb01 and ShAb02 provided significant protection in comparison to the isotype control. In this model, ShAb01 provided the greater protection with delayed onset of illness, compared to the ShAb02 or isotype control group as indicated by weight loss (Fig. 3b), and milder clinical manifestations (Fig. 3c). The survival rate was 86% for the ShAb01 group, compared to 43% for the ShAb02 group and 0% for the control group, where all mice had either succumbed to the infection or were euthanized by day 8 post-challenge (Fig. 3d). The two ShAb groups were statistically equivalent to each other, while the ShAb01 group was significantly different than the isotype control group. The protective effect of ShAb01 was further highlighted by undetectable viral load in lung bronchoalveolar lavage (BAL) fluid samples at 2 days post infection, while the ShAb02-infused animals had an approximately ten-fold lower viral load than the isotype control group (Fig. 3e).

### Structure of ShAb01 and ShAb02 in complex with SARS-CoV-2 RBD

**Fig. 4:**
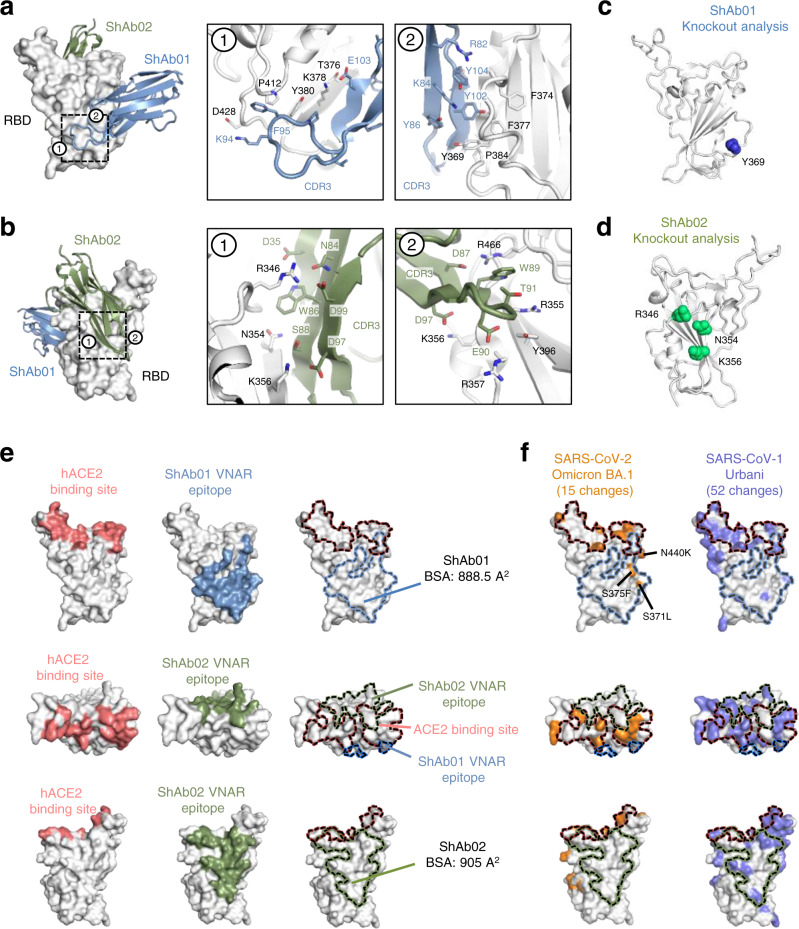
Structural analysis of the interaction of ShAb01 and ShAb02 with SARS-CoV-2 RBD. **a** Crystal structure of ShAb01 and ShAb02 VNARs in complex with SARS-CoV-2 RBD. The critical residues of ShAb01-RBD interaction are shown in two zoom-in panels, with extensive contacts made by the CDR3 loop. **b** Crystal structure of the ShAb01-ShAb02-RBD complex rotated from **a**. The critical residues of ShAb02-RBD interaction are shown in two zoom-in panels, with extensive contacts made by the VNAR CDR loops 1–3. **c** Epitope mapping by alanine mutagenesis scanning analysis identified SARS-CoV-2 RBD residue Y369 as a critical contact residue for ShAb01 binding. **d** Epitope mapping by alanine mutagenesis scanning analysis identified SARS-CoV-2 RBD residues R346, N354, and K356 as critical contact residues for ShAb02 binding. **e** Footprint of ACE2, ShAb01, and ShAb02 shown in multiple orientations. Overlay of the footprints are shown as an enclosed dashed line. SARS CoV-2 RBD is shown as white surface presentation; ACE2, ShAb01, and ShAb02 footprints on RBD are colored with salmon, green and slate respectively. **f** Footprint of ACE2, ShAb01 and ShAb02 footprints on SARS-CoV-2 WA-1 RBD. SARS-CoV-2 Omicron BA.1 and SARS-CoV-1 Urbani residue differences are highlighted in orange and purple, respectively.

To understand the molecular interactions of the shark VNARs with SARS-CoV-2 spike RBD, we carried out structural and mutagenesis studies. We determined the crystal structure of SARS-CoV-2 WA-1 RBD in complex with ShAb01 and ShAb02 VNARs at a resolution of 2.5 Å by X-ray crystallography (Fig. 4a, b). The structures of ShAb01 and ShAb02 show that they are both VNARs with typical β-sheet topology and an extensive RBD-contacting interface (Supplementary Fig. 3). ShAb01 binds to the RBD with a total buried surface area (BSA) of 888.5 Å^2^ primarily engaging the CDR3 loop (728.5 Å^2^) and the first β-sheet recognizing one side of the SARS-CoV-2 RBD, reminiscent of CR3022 binding and distal from the ACE2 binding site (Fig. 4a, e, Supplementary Tables 4, 6). Within the CDR3 loop, a β-sheet (G100-E104) forms an anti-parallel interaction with an RBD β-sheet (S375-S379), and the CDR3 tip (Y86-G100) interacts with multiple loops of the RBD including Y369-A372, F374-T385, A411-Q414, and D427-F429. In addition, the N-terminal residues of ShAb01 bind to a pocket located between RBD residues D405-A411 and β-sheet S375-Y380 thus increasing the overall ShAb01-RBD interaction.

ShAb02 binds to the opposite face of the SARS-CoV-2 RBD in relation to the ShAb01 epitope (Fig. 4b, e, Supplementary Tables 5, 7). ShAb02 forms multiple interactions with the RBD using CDR1 (151.5 Å^2^), HV2 (146.5 Å^2^), and CDR3 (533 Å^2^) with a total BSA interface of 905 Å^2^. The major contact region between ShAb02 and RBD is facilitated through four negatively charged residues of the ShAb02 CDR3 loop. Notably, the SARS-CoV-2 RBD R346 forms a hydrogen bond with ShAb02 Y101, a salt bridge with ShAb02 D99, and pi-pi interactions with ShAb02 W86. In addition to the CDR3 loop, ShAb02 CDR1 and HV4 engages RBD residues I468-E471 through main chain interactions, while the ShAb02 HV2 loop uses main chain atoms to engage RBD residues G447-N450 (Supplementary Fig. 3).

Alanine scanning mutagenesis of the RBD was used to identify amino acids critical for binding (Fig. 4c, d). The analysis uncovered Y369 as a critical residue for ShAb01-RBD recognition, and R346, N354, and K356 as critical residues for ShAb02-RBD recognition (Fig. 4c, d). The RBD Y369 engages a hydrophobic pocket created by the ShAb01 residues, G100, Y102, and Y86, and stabilizes the antiparallel β-sheet interaction between the RBD and ShAb01. In addition, the RBD R346 residue is a point of multiple interactions between the RBD and ShAb02. RBD residues N354 and K356 insert into a structural groove formed by two ShAb02 CDR3 β-sheets to create extensive buried surface interaction.

Analysis of the binding epitopes with the ACE2 binding site shows minimal overlap for either ShAb01 or ShAb02 VNAR (Fig. 4e). However, further binding studies using the VNAR domains alone (with Fc domains removed) showed that the ACE2 blocking activity of ShAb01 was maintained, indicating that despite only minor overlap with the ACE2 binding site, this was sufficient to provide robust blocking (Supplementary Fig. 9).

**Fig. 5:**
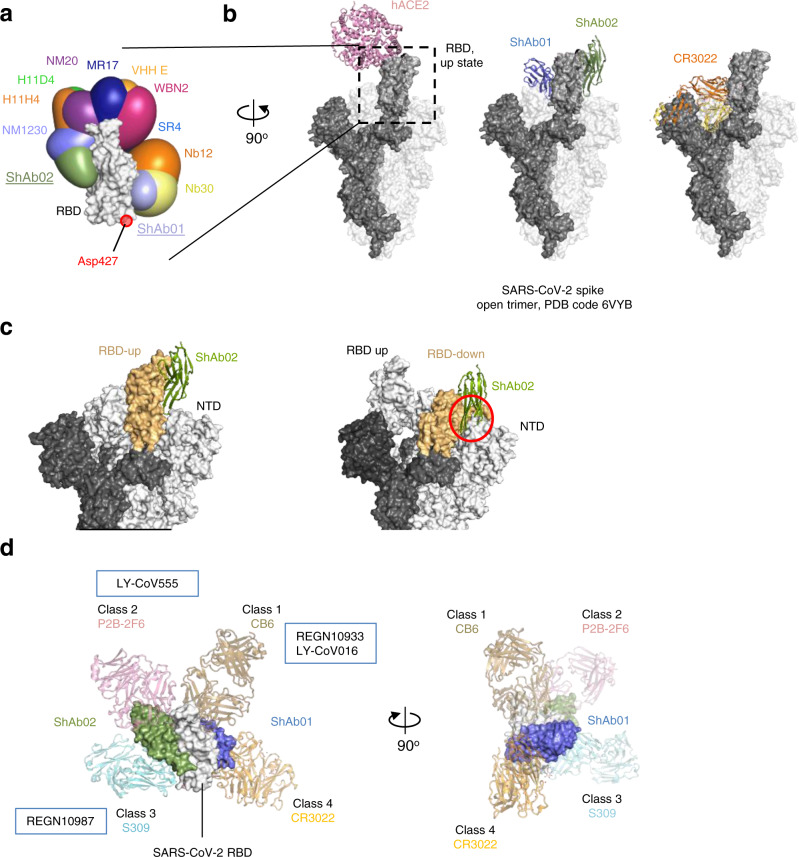
Crystal structure of ShAb01 and ShAb02 in the context of prefusion S. **a** SARS-CoV-2 RBD (light gray) is shown in surface representation, with ShAb01, ShAb02, and 10 other nanobodies overlaid and shown in smooth surface representation. **b** Structure alignment of the ShAb01-ShAb02-RBD complex (center) to a partially open S-2P structure (1-RBD-up conformation, PDB ID:6VYB). Both ShAb01 and ShAb02 have full access to their respective epitopes in this structure. ACE2 (left) and CR3022 (right) are shown for reference. **c** The SARS-CoV-2 spike is displayed as in b with the RBD in wheat color and ShAb02 (green) aligned with (left), RBD-up conformation and (right) RBD-down conformation. The clash of ShAb02 and NTD in the RBD-down conformation is indicated by the red circle. **d** Structure of ShAb01-ShAb02-RBD complex (surface representation) overlaid on previously reported antibody-RBD complex structures (representing frequently observed antibody recognition classes). mAbs CB6, P2B-2F6, S309, and CR3022 are shown in cartoon representation and colored sand, pink, cyan, and orange respectively.

Numerous monoclonal antibodies (mAbs) and nanobodies have been identified that can neutralize SARS-CoV-2^22–34^. We compared the ShAb01 and ShAb02 binding epitopes to all previously described mAbs and nAbs (with the closest epitopes shown for comparisons). ShAb01 binds to the same face of the RBD as mAb CR3022 and falls into the Class-IV grouping of RBD-targeting antibodies^35,36^. The ShAb01 epitope overlaps with other nanobody epitopes, but the ShAb01 epitope is more extensive, and extends below the typical Class-IV epitope, with the ShAb01 CDR3 interacting with residues proximal to D427, typically not seen with other nanobodies (Fig. 5a and Supplementary Fig. 4). Compared with nanobodies WNB10, NB30, VHH V, and 3B4, which have significant epitope overlap^25,27,32^, the extended CDR3 loop of ShAb01 expands the binding epitope to a highly sequence-conserved region of the RBD (Supplementary Fig. 5).

ShAb02 binds to the face of the RBD molecule that is targeted by antibodies designated as Class-III, in common with the COVID-19 emergency-use-authorized mAbs S309 and REGN10987 (Fig. 5b–d). The ShAb02 molecule is incompatible with binding to the closed S conformation due to steric clashes with a neighboring NTD (Fig. 5c). Comparison of ShAb02 with other nanobodies (Supplementary Figs. 4, 5) indicates that the ShAb02 epitope is unique among nanobodies described here and elsewhere. Most camelid nanobodies bind to a region above the ShAb02 epitope, closer to the ACE2 binding site (Fig. 5b and Supplementary Fig. 4)^23,25,30,31^. The 3B4 and 2C02 nanobodies recently identified from a synthetic VNAR library^33^ have the most similar epitopes to ShAb01 and ShAb02 respectively. VNAR 3B4 overlaps with the ShAb01 epitope and also forms a salt-bridge with D427. VNAR 2C02 also has an overlapping epitope with ShAb02, but both molecules have low sequence similarity, and in the case of 2C02 an altered ~15-degree vertical tilt in binding angle (Supplementary Fig. 4c). Sequence analysis of related sarbecovirus RBDs indicates that sequence variation within the ShAb02 epitope is minimal for SARS-CoV-2 viral variants including Omicron subvariants but is notable for other sarbecoviruses including SARS-CoV-1 (Supplementary Fig. 5).

Analysis of the structure of the Omicron BA.1 RBD in comparison allows a clear understanding of how the ShAb01 binding is reduced (Supplementary Fig. 6). The Omicron BA.1 S371L and S375F mutations are within the ShAb01 epitope, while the S373P variation causes a rotation of the helix 361–371 which is altered in location by ~3–6 Å. Y369 is rotated by 180° in the Omicron RBD structure and loses a pi-pi interaction with ShAb01 Y102. This structural change further places the S373L and S375F in locations that would readily clash with ShAb01 Y102 and Y104 respectively, further disrupting the ShAb01 binding.

### Multi-specific molecules combining ShAb VNARs show increased anti-SARS-CoV-2 activity

**Fig. 6:**
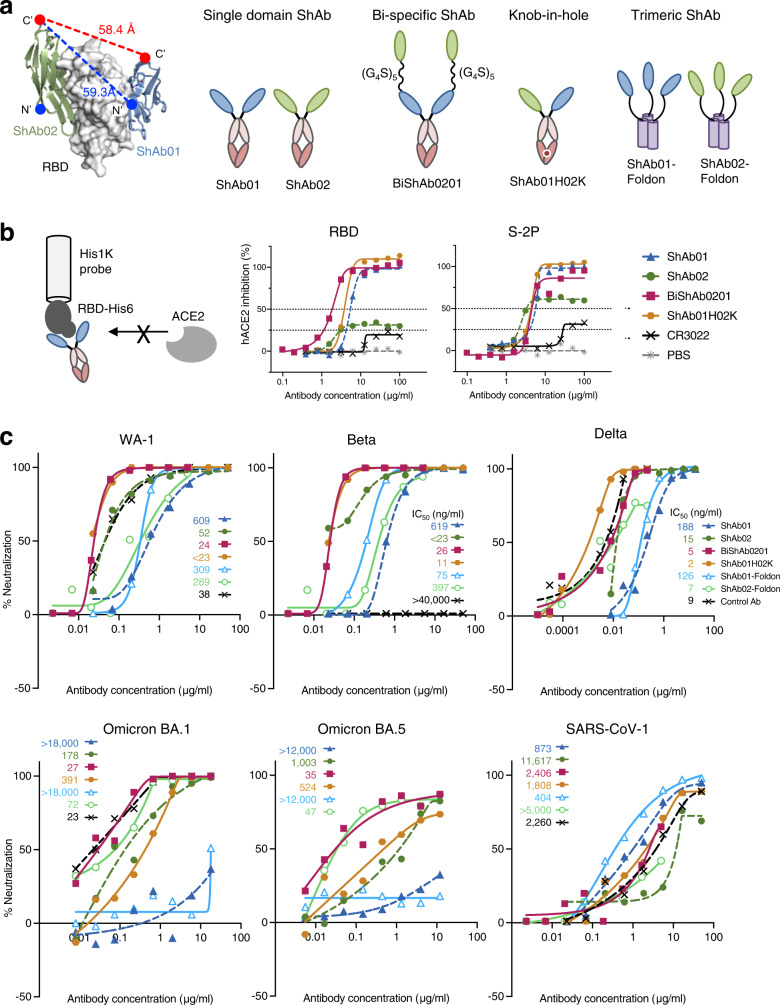
Structure-based design of multi-domain ShAb molecules. **a** Left, SARS-CoV-2 RBD is shown in surface representation with ShAb01 and ShAb02 VNARs in ribbon representation. The distance from the ShAb02 VNAR C-terminus to either the N- or C-terminus of ShAb01 is shown. Right, design schematics of multi-specific molecules BiShAb0201, and ShAb01H02K ‘knob-in-hole’ constructs, and trivalent ShAb-Foldon constructs. **b** BLI measurement of ACE2-inhibition to SARS-CoV-2 WA-1 RBD (center) and S-2P (right). The measurements are performed with immobilized SARS-CoV-1 RBD, with ShAb molecules and ACE2 in solution. **c** Neutralization of SARS-CoV-2 WA-1, Beta, Delta, Omicron BA.1, Omicron BA.5, and SARS-CoV-1 Urbani pseudoviruses by ShAb01, ShAb02, ShAb01H02K, BiShAb0201, ShAb01-Foldon and Shab02-Foldon. P2B-2F6 was used as a control antibody for SARS-CoV-2 strains except for Delta and Omicron BA.1 where COV2-2196 was used, and CR3022 was used for SARS-CoV-1. IC_50_ values for pseudovirus neutralization, are shown in ng ml^−1^, for each virus and ShAb or control mAb, with SD indicated by vertical lines. Source data are provided as a Source Data file.

Given the non-overlapping nature of the ShAb01 and ShAb02 epitopes, we explored various designs to engineer combined molecules that can provide increased breadth and potency into a single immunotherapeutic. Using the structural information, we analyzed the distances between the N- and C-termini of the VNAR molecules (Fig. 6a, Supplementary Fig 7a) to determine the required spacing or linker lengths, to enable simultaneous binding by each VNAR to a single RBD molecule in the context of multi-domain molecules. We then produced a set of multi-domain molecules containing ShAb01, ShAb02 or both VNARs (Fig. 6a, and Supplementary Fig. 7) for further assessment.

One of the bispecific designs, ShAb01H02K, combined a ShAb01-Fc and a ShAb02-Fc by using knob and hole mutations in the Fc regions to generate a heterodimeric antibody (Fig. 6a, Supplementary Fig. 7b). In another design, designated BiShAb0201, the ShAb01 and ShAb02 domains were added to the Fc region sequentially, connected by a long flexible 25-amino acid linker (Fig. 6a, Supplementary Fig. 7c). The ShAb02-ShAb01 sequential order of this construct was chosen based on the relative position of the N- and C-termini of the VNARs when bound to the RBD. Reversing the order would have required an even longer linker to traverse the distance between the C-terminus of VNAR01 and the N-terminus of VNAR02 (Supplementary Fig. 6). A third design generated trimeric molecules by fusing each of the ShAb VNARs to a Foldon trimerization domain^37^ (Fig. 6a, Supplementary Fig. 7d). This resulted in trivalent rather than bivalent binding by the respective ShAbs, although these molecules lack the Fc fragment so they can only act as neutralizing compounds without the added benefit of effector functions.

ShAb01H02K and BiShAb0201 showed large improvements in antigen-affinity (Supplementary Figs. 2, 8 and Supplementary Table 8). Measurement of binding parameters (K_D_) against RBD molecules by biolayer interferometry revealed that the bispecific proteins had > 10-fold increased affinity for all SARS-CoV-2 VoC RBDs over ShAb01 or > 3-fold increased affinity over ShAb02 (Supplementary Fig. 8). Affinity of both ShAb01H02K, and BiShAb0201 for the SARS-CoV-2 Delta RBD was remarkably 389,000-fold and 149,000-fold higher than ShAb01 and ShAb02 respectively, which is due to an exceptionally slow off rate (Supplementary Figs. 2, 8a, Supplementary Table 8). In the case of Omicron BA.1 RBD, ShAb01 lost much of its binding as a result of the repositioning of Y369 within the RBD (Fig. 2 and Supplementary Fig. 6), but this could be rescued when combining ShAb01 with ShAb02 in the context of BiShAb0201 and ShAb01H02K (Supplementary Fig. 8a). These bispecific molecules also showed increased affinity by 1.6–1.9-fold to SARS-CoV-1 RBD over ShAb01 even though ShAb02 showed no binding to SARS-CoV-1 RBD as a monospecific molecule.

In two BLI-based ACE2 competition assay formats, using either RBD or S-2P as the target, both ShAb01H02K and BiShAb0201 blocked ACE2 binding by S-2P, at similar levels to ShAb01. Moreover, BiShAb0201 was able to inhibit ACE2 binding to RBD better than either ShAb01 or ShAb02 molecules (Fig. 6b).

The pseudovirus neutralization potency of these multi-specific molecules was compared directly between the parental ShAb01 and ShAb02, and the four combination molecules (ShAb01H02K, BiShAb0201, ShAb01-Foldon, and ShAb02-Foldon. (Fig. 6c). ShAb01H02K and BiShAb0201 display IC_50_ pseudovirus neutralization levels of < 25 ng ml^−1^ for most SARS-CoV-2 variants, which represent a 18–667-fold increase compared to ShAb01, and up to a 28-fold increase compared to ShAb02 alone (Supplementary Fig. 8b). In the case of the Omicron variants tested, the BiShAb0201 molecule neutralized with an IC_50_ of 27 or 35 ng ml^−1^ against BA.1 and BA.4/5 variants respectively, which was considerably improved over the ShAb02 neutralization levels. Among the trimerized ShAbs, ShAb01-Foldon, had slight increases in the neutralization titers against SARS-CoV-2 WA-1, Alpha, Beta, and Delta pseudoviruses. While for the Omicron variants assessed, the neutralization IC_50_ titers were 72 and 47 ng ml^−1^ for the ShAb02-foldon molecule. In the case of SARS-CoV-1 neutralization potency, there were modest if any improvements in neutralization by the bispecific ShAbs over the parental ShAb molecules. ShAb01 had an IC_50_ of 873 ng ml^−1^, which improved to 404 ng ml^−1^ in the case of the ShAb01-Foldon molecule (Fig. 6c and Supplementary Fig. 8b).

To further dissect the neutralization potency of the nanobodies in their various configurations we compared the VNAR molecules (VNAR01, VNAR02, and VNA0201) to their bivalent Fc-containing counterparts (Supplementary Fig. 9). The VNARs were obtained by proteolytic digestion using an encoded human rhinovirus-3c protease site between the VNAR and the Fc domain (Supplementary Fig. 9a). The ACE2 binding profiles of the VNAR molecules is similar to their Fc-containing counterparts (Supplementary Fig. 9b). The VNARs also show neutralization potencies that are comparable to their bulkier counterparts (Supplementary Fig. 9c, d). It is worth noting that when comparing the proteins in terms of molar concentration and avidity potential, a VNAR molecule has one binding moiety, while the ShAbs and BiShAb0201 have two and four binding moieties per molecule, respectively.

**Fig. 7:**
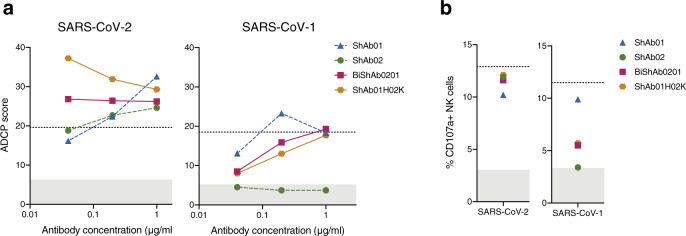
Fc-mediated effector functions by ShAb molecules. **a** ADCP assay using SARS-CoV-2 WA-1 or SARS-CoV-1 Urbani S-2P-labelled beads incubated with phagocytic cells. ADCP scores measure the relative uptake of the bead-labeled proteins. Dotted black line indicates positive control (CR3022), and gray shaded area indicates positivity cutoff as determined by an irrelevant anti-Zika virus antibody (MZ4). **b** NK cell degranulation in the presence of ShAbs and CoV S-2P. The dotted black line indicates positive control (1:1 mixture of WRAIR-2039 and WRAIR-2123 antibodies for SARS-CoV-2, and CR3022 for SARS-CoV-1). The gray shaded area indicates positivity cutoff as determined by an irrelevant anti-Zika virus antibody (MZ4). Source data are provided as a Source Data file.

The ShAb01, ShAb02, and the bispecific combinations (BiShAb0201 and ShAb01H02K), were tested in cell culture experiments to assess their ability to exert Fc-dependent effector functions (Fig. 7). When ShAb molecules were incubated with bead-labeled S-2P protein from the SARS-CoV-1 Urbani, or SARS-CoV-2 WA-1 strains, and added to phagocytic cells, they generated antibody-dependent cellular phagocytosis (ADCP) at high levels (Fig. 7a). In this context, the combination ShAbs mediated greater ADCP activity than either ShAb01 or ShAb02 alone. ShAb01 and the ShAb bispecific molecules showed strong ADCP activity against SARS-CoV-1, while ShAb02 showed no activity.

In NK cell activation experiments, co-incubation of ShAbs with immobilized WA-1 S-2P was followed by addition of NK cells. Degranulation was then assessed by a CD107a capture assay (Fig. 7b). Against SARS-CoV-2, all the ShAb molecules, including the bispecific molecules showed levels of degranulation that were slightly lower, but comparable to the positive control which was a mixture of antibodies WRAIR-2039 and WRAIR-2123^34^. Using SARS-CoV-1 S-2P, ShAb01 produced a similar response (10%) compared to the control antibody CR3022 (11.5%) while the bispecific ShAbs had a 6% response, ShAb02 showed no NK degranulation activity against SARS-CoV-1.

### Breadth of reactivity of ShAb molecules

**Fig. 8:**
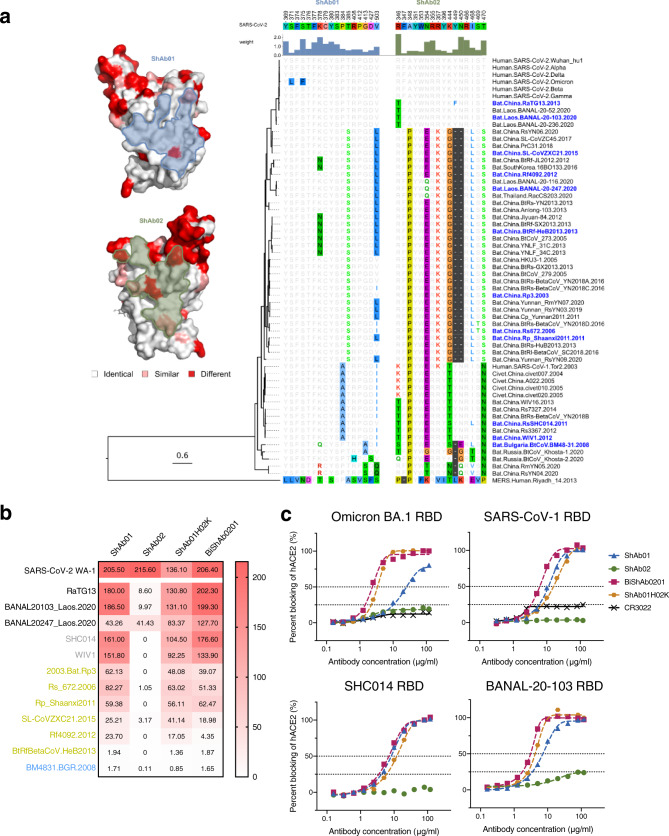
ShAb molecules show activity against diverse sarbecoviruses. **a** Epitope conservation and cross-reactivity analysis. Highly conserved RBD residues are colored in white, similar residues are colored in pink, and differing residues are colored in red. ShAb01 and ShAb02 epitopes are shown as a light blue and light green surface respectively. The epitope residues are numbered according to the Wuhan reference strain; the strength of the interaction between the ShAb and RBD is indicated by the height of the histogram bars above the sequence alignment (right). Sequences are ordered by phylogenetic relationships based on a maximum likelihood phylogenetic tree derived from RBD amino acid sequences. **b** BLI measurement of ShAb molecules binding to various sarbecovirus RBDs, (clade 1b (black), clade 1a (gray), clade 2 (gold) and clade 3 (blue)). Heat map shows area under curve (AUC) values. RBD molecules were immobilized to the probe with ShAb molecules in solution. **c** ACE2-inhibition by ShAbs to SARS-CoV-2 Omicron BA.1 RBD, SARS-CoV-1 RBD, and two ACE2-utilizing sarbecovirus RBD molecules. The measurements are performed with immobilized RBD, and with ShAbs and ACE2-Fc in solution. CR3022 was used as a control. Source data are provided as a Source Data file.

Given the ability of ShAb01, ShAb02, and the multi-specific ShAb molecules to recognize all SARS-CoV-2 VoC, and other bat-derived sarbecoviruses (Fig. 2d), we assessed the binding epitopes of ShAb01 and ShAb02 for structural and sequence conservation (Fig. 8a). The epitope of ShAb01 is well conserved between clade 1a and 1b strains e.g., SARS-CoV-2 and SARS-CoV-1, with minimal sequence differences. For the more distantly related sarbecoviruses from clade 2 and clade 3, the K378N/Q residue difference between SARS-CoV-2 is a significant difference at a critical residue within the ShAb01 epitope. In the case of the ShAb02 epitope, there is complete conservation across SARS-CoV-2 VoC, while significant changes are observed for most other sarbecoviruses even the highly related clade 1b RaTG13 strain where a R346T difference is seen. Given the significant role that R346 provides in ShAb02 recognition, this residue could readily facilitate viral escape. We further assessed the binding of ShAb01, ShAb02, ShAb01H02K, and BiShAb0201 against a panel of 12 sarbecovirus RBD molecules from sarbecovirus clades 1a, 1b, 2, and 3. ShAb01 showed strong binding to 7, intermediate binding to 3, and no binding to 2 of the 12 RBD molecules. The two RBDs that did not show binding were from BtRfBetaCoV.HeB2013 or BM4831.BGR.2008 strains containing N378 and Q378 respectively. In the case of ShAb02, binding was largely absent except for the RBD from BANAL20247_Laos_2020 strain. This virus is a clade 1b sarbecovirus with high sequence similarity to SARS-CoV-2. The multi-specific antibodies showed a similar pattern of binding as seen for ShAb01, with BiShAb0201 in general having higher binding levels than ShAb01H02K. We further assessed whether the ShAb molecules could provide ACE2 blocking activity against clade 1a and 1b sarbecovirus RBDs with high ACE2 binding. In general, ShAb01, BiShAb0201, and ShAb01H02K showed robust ACE2 blocking activity similar to that seen with SARS-CoV-2 WA-1 strain, while BiShAb0201 had marginally higher activity in all cases. For the SARS-CoV-2 Omicron BA.1 RBD, the ShAb01 had reduced ACE2 blocking activity in line with the reduced binding and neutralization activity (Fig. 6c).

## Discussion

The SARS-CoV-2 pandemic continues to cause significant upheaval due to the continued emergence of novel VoC that have evolved to be more transmissible and evade prior immunity. This has resulted in modified immunization schedules for fully licensed and EUA vaccines, and in the case of many therapeutic mAbs, removal of their EUA status in the US, largely due to inactivity against the Omicron sublineages. Here we detail the elicitation of a set of nanobodies in nurse sharks by immunization, followed by bio-panning to identify high affinity binders against SARS-CoV-2. IFA/CFA adjuvants in combination with three separate immunization schedules were utilized to maximize the immune response in this animal model. Nurse sharks immunized with SARS-CoV-2 RBD yielded the prototypic ShAb01 and ShAb02 VNAR molecules after 3 or 4 immunizations. In addition, we immunized two nurse sharks with a RFN nanoparticle, which required four immunizations to generate sufficient immune titers against SARS-CoV-2. Immunization with the SpFN generated a robust immune response after three immunizations and allowed identification of multiple unique clones. The elicitation of a robust immune response by SpFN matches studies carried out in non-human primates^17,18^, and SpFN is currently under assessment in a human phase I clinical trial (NCT04784767).

The shark VNAR domains were prepared with a human Fc domain for further characterization and were divided into two antigenic groups - exemplified by ShAb01 and ShAb02 - with all nanobodies displaying high affinity to SARS-CoV-2 RBD. A major distinction between the two antigenic groups, was ACE2 blocking capacity within the ShAb01 group, while both ShAb01 and ShAb02 had potent neutralization against the SARS-CoV-2 WA-1 strain and viral VoC. In the case of ShAb02, the potent neutralization included VoC Omicron BA.1, while VoC Delta IC_50_ neutralization levels were seen at a level of 15 ng/ml. While ShAb01 did not neutralize Omicron BA.1 or BA.5, it did display strong binding to clade 1a sarbecoviruses including SARS-CoV-1, and neutralization of SARS-CoV-1 at levels comparable to SARS-CoV-2 variant neutralization levels, indicative of a common and similar epitope being targeted.

Given the potent neutralization levels, we further assessed ShAb01 and ShAb02 in the K18-hACE2 transgenic mouse model. For both ShAbs, we observed significant protection against disease and death in this model. ShAb01 showed greater protection than ShAb02 at the concentration used (equivalent to 10 mg/kg), particularly highlighted by undetectable levels of virus in BAL fluid at 2 days post challenge. Passive transfer of ShAb02 also provided protection, but not to the level seen with ShAb01. This is despite ShAb02 having greater neutralizing potency against SARS-CoV-2 WA-1. ShAb01 does provide robust ACE2 blocking activity in both the context of RBD- or S-based assays, and this additional property may explain the differences seen in the challenge study.

To understand the nanobody recognition properties, we determined the structures of both ShAb01 and ShAb02 in complex with RBD. The non-overlapping epitopes recognized by ShAb01 and ShAb02 broadly fall into previously described RBD class 4 and class 3 epitopes, exemplified by mAbs CR3022 and S309 respectively, and the RBD of the S would be required to adopt the “up” conformation to enable ShAb binding. When the RBD is in the “down” conformation, there is steric interference with an NTD from an adjacent protomer. In contrast to most potent neutralizing antibodies, the ShAb epitopes had minimal overlap with the ACE2-binding site. The ShAb molecules target two highly conserved faces of the RBD which matches to the broad binding and neutralization activity that was observed. ShAb01 and ShAb02 utilize the CDR3 loop in a side-to-side binding of the SARS-CoV-2 RBD, contrasting with typical CDR loop tip interactions seen with camelid-derived nanobodies and antibodies from humans or other animals. This side-to-side binding is also seen with a set of nanobodies identified from engineered cartilaginous fish VNAR phage-display libraries^33^.

The unique interaction pattern of the two ShAbs on two opposing faces of the RBD molecule, in a sandwich-like complex, was leveraged for structure-based multi-specific designs. By coupling the two epitope specificities into single molecules, we could assess the combination of complementary targeting, neutralization, and ACE2 blocking activity. The multi-specific molecules did display improved activity over the parent ShAb molecules, and as novel VoC emerged and were assessed, both ACE2 blocking activity and neutralization potency were maintained. In addition, ADCP activity against both SARS-CoV-2 and SARS-CoV-1 was preserved in the multi-specific molecules. In the case of NK cell degranulation, both BiShAb0201 and ShAb01H02K generated robust activity in the presence of SARS-CoV-2 S, while activity in the presence of SARS-CoV-1 S was reduced in comparison to ShAb01. Fc-mediated functions are important for the anti-viral activity of some neutralizing mAbs against SARS-CoV-2^34,38,39^. In addition, further multimerization of the VNAR domains or combination with Fc modifications are likely to further increase the activity and therapeutic half-life of these nanobodies. The observation that the VNAR domains by themselves, or in the Fc-fused configurations, and as multi-specific molecules, were able to potently neutralize a panel of SARS-CoV-2 viral VoC as well as SARS-CoV-1 demonstrates the versatility of these molecules. Therapeutic formulations relying on easy and accessible delivery options, such as aerosolization, could take advantage of the small size of the VNARs. Alternatively, when used as ShAbs or BiShAbs, in addition to neutralization, the ability to utilize the Fc-dependent immune functions further increases their therapeutic potential.

Given the binding and neutralization data, we further assessed the ShAb molecules against a more diverse panel of sarbecovirus clade 1–3 derived RBD molecules for both binding and ACE2 blocking activity. In large part, the biochemical activity matched to the epitope sequence conservation, with RBDs from clade 1a, 1b, and 2 having strong binding to ShAb01, BiShAb0201, and ShAb01H02K molecules, while ShAb02 did not display any binding activity for non-clade 1b RBDs. Within the ShAb01 epitope, RBD differences seen amongst sarbecoviruses (Fig. 8a) such as N/Q378 likely reduce binding and limit the breadth of this nanobody. However, variations at this position have not been seen in clade 1b viruses (SARS-CoV-2-related), and reduction in ShAb01 binding with a K378A mutation assessed as part of the alanine scanning studies has not been observed.

The immunization of nurse sharks to identify unique antiviral compounds has previously been described for Ebola virus^40^, while the nanobodies described in this study firmly establish that CoV-recognition molecules with high stability can be readily elicited by vaccination. The use of cartilaginous fish VNAR phage-display libraries^35^ or the analogous llama immunization and/or camelid based libraries^25^ are complementary strategies that serve to identify clones with potent neutralization properties. Structure-based design allowing combination of desirable features of ShAb01 and ShAb02 allowed generation of high affinity multi-specific molecules that are efficacious against SARS-CoV-2 variants and other sarbecoviruses. The combination of highly stable and minimal-sized molecules with exceptional antiviral functions can provide greater capacity to resist SARS-CoV-2 viral variation and possibilities for development for future diagnostic, therapeutic or prophylactic use.

## Methods

### Immunogen design and production

Following release of the SARS-CoV-2 sequence on Jan 10^th^, 2020, initial RBD, RBD-Ferritin (RFN) and Spike-Ferritin (SpFN) immunogens were designed. Subsequent iterative immunogen design and optimization utilized atomic models of the SARS-CoV-2 RBD molecule, or the SARS-2 spike trimer structure PDB ID: 6VXX, and PDB ID: 3BVE for the *Helicobacter pylori* Ferritin, and PDB ID: 4LQH for the bullfrog linker sequence. Pymol 2.3.2 (Schrödinger) was used to generate the ferritin 24- subunit particle, and a map created in UCSF Chimera (Pettersen et al., 2004) was supplied to cisTEM (Grant et al., 2018) “align_symmetry” to align the ferritin particle to an octahedral symmetry convention. This was supplied to “phenix.map_symmetry” to generate a symmetry file and PDB file, for octahedral (for RBD-fusions) and D4 (for trimer-fusions) symmetry. Spike-domain ferritin nanoparticle fusions were modelled using Pymol and Coot 0.8.9.2 (Emsley et al. 2010) and expanded using “phenix.apply_ncs” (Liebschner et al., 2019). Visual analysis and figure generation was conducted using ChimeraX 1.1 and PyMOL 2.3.2.

RBD-Ferritin designs were generated by assessment of the hydrophobic surface of the SARS-CoV-2 RBD surface and determining surface accessible mutations that reduced the hydrophobic surface. Spike-Ferritin designs were created by modeling the coiled-coil region between Spike residues 1140 and 1158 and increasing the coil-coil interaction either by mutagenesis, or by increasing the length of the interaction region.

### Shark immunizations

All research in this study involving animals was conducted in compliance with the Animal Welfare Act, and other federal statutes and regulations relating to animals and experiments involving animals and adhered to the principles stated in the Guide for the Care and Use of Laboratory Animals, NRC Publication, 1996 edition. The sharks used in this study were acquired under a Special Activity License granted by the Florida Fish and Wildlife Conservation Commission. Sharks were sedated with MS-222 prior to any procedure and all animal procedures were conducted under protocol #0318003 in accordance with University of Maryland, School of Medicine Institutional Animal Care and Use Committee (IACUC)- and USAMRDC Animal Care and Use Review Office (ACURO)-approved protocols.

Six juvenile nurse sharks (two males and four females, aged between 2–3 years and weighing between 1.8 and 3.8 kg (Supplementary Table 1)), were held in a continuously-recirculating 12,000 L seawater tank maintained at 28 °C, in the Aquaculture Research Center at the Institute of Marine & Environmental Technology (IMET), Baltimore, USA. Following a sufficient period of acclimatization, animals were primed with 200–250 μg antigen emulsified in complete Freund’s adjuvant (CFA) administered subcutaneously into the ventral surface of the lateral fin. At approx. 4-week intervals sharks were boosted, first with 200–250 μg antigen emulsified in incomplete Freund’s adjuvant (IFA) administered subcutaneously into the ventral surface of the lateral fin, then intravenously with 100 μg antigen diluted in shark-modified PBS (unadjuvanted) and administered directly into the caudal sinus. Sharks ‘Pink’ and ‘Red’ were immunized four times with SARS-CoV-2 RBD at week 0, 4, 8, and 13; ‘Green’ and ‘Yellow’ were immunized four times with SARS-CoV-2 RBD-Ferritin (RFN) at week 0, 4, 8, and 36; ‘Purple’ and ‘Blue’ were immunized three times with SARS-CoV-2 Spike-Ferritin (SpFN) at week 0, 4, and 8 (Supplementary Table 1). Small bleeds were drawn 2 weeks after each immunization, mixed with 1/10 volume of heparin (reconstituted to 1000 U/ml in shark-modified PBS), and centrifuged at 115 g for 10 min to separate peripheral blood lymphocytes (PBLs) and blood plasma. Plasma samples were tested for the presence of antigen-specific IgNAR by binding ELISA as previously detailed^41^, but with the following changes: 96-well Nunc MaxiSorp flat bottom microtiter plates were coated with recombinant SARS-CoV-2 RBD or stabilized trimeric spike protein (S-2P) diluted in PBS to 1 μg/ml. Plates were incubated overnight at 4 °C then blocked with MPBS (PBS containing 5% milk powder) at room temperature for 2 h; control wells were blocked without prior antigen coating. The mouse mAb GA8 was used to detect IgNAR binding. Once target-specific IgNAR titers had reached sufficient levels a larger blood sample was taken from the animal, and PBLs harvested for RNA isolation.

### VNAR library construction

VNAR libraries were built for the RBD-immunized animals, Pink and Red, using PBLs harvested at bleed 3 (week 10) then at bleed 5 (week 15); from the RFN-immunized animals, Yellow and Green, using PBLs harvested at bleed 4 (week 10) then at bleeds 5 and 6 (weeks 30 and 40). A single library was built from each SpFN-immunized animal, Blue and Purple, utilizing PBLs harvested at bleed 4 (week 10).

PBLs were lysed in phenol solution and total RNA prepared from each as per standard protocols. Oligo-dT-primed cDNA was prepared and used as the template for PCR amplification of IgNAR variable regions (VNARs) with the handled primers NARFr4-Rev1 (5’-ATA ATC AAG CTT GCG GCC GCA TTC ACA GTC ACG ACA GTG CCA CCT C-3’) and NARFr4-Rev2 (5’-ATA ATC AAG CTT GCG GCCGCA TTC ACA GTC ACG GCA GTG CCA TCT C-3’) mixed in an equal ratio, and NARFr1-For (5’-ATA ATA AGG AAT TCCATG GCT CGA GTG GAC CAA ACA CCG-3’). The ~400 bp PCR products were cleaned, digested overnight with the restriction enzymes NcoI and NotI at sites introduced in the primer handles, cleaned again then cloned into similarly cut, shrimp alkaline phosphatase-treated pHEN2; this phagemid vector has a bacteriophage packaging signal and produces soluble VNAR fused to the phage gene III coat protein thus physically linking the VNAR sequence with its antigen binding ability. The resultant VNAR libraries were phenol:chloroform cleaned, resuspended in 10 μl DEPC-treated water, and transformed into electrocompetent *E. coli* TG1 cells (Agilent). Cells were resuspended in 2xTY media and allowed to recover for 1 h at 37 °C then plated on TYE agar bioassay plates containing 100 μg/ml ampicillin (A100) and 2% glucose (G2). Each of the VNAR libraries produced exceeded 10^10^ members in size. Colonies were scraped from the bioassay plates into 2xTY/A100/G2 media containing 30% sterile glycerol and aliquots of the library flash frozen for storage at −80 °C.

### VNAR library selections

Library selections were performed as previously described^11^. Briefly, a single aliquot of library stock was added to pre-warmed 2×TY/A100/G2 and grown with shaking at 37 °C to mid-log phase prior to infection with M13K07 helper phage. Cultures were spun and cell pellets resuspended in 2xTY containing 100 µg/ml ampicillin, 50 µg/ml kanamycin, and 0.2% glucose (2xTY/A100/K50/G0.2) then incubated with shaking at 30 °C overnight to permit library expression. Phage were precipitated from the culture supernatant by the addition of 1/3 volume of PEG-NaCl and phage pellets resuspended in PBS and titered ready for use in panning.

Libraries were panned on immunotubes (Maxisorp, Nunc) coated overnight at 4 °C with antigen diluted in PBS to the required concentration, then blocked with 5% MPBS. Selection was conducted by incubating coated immunotubes for 2 h at room temperature with 1 ml of phage solution in 4 ml of 5% MPBS. Following incubation unbound phage were discarded, the immunotube washed, then bound phage eluted with 1 ml of 100 mM triethylamine. The phage solution was neutralized by the addition of 0.5 ml of 1 M Tris-HCl, pH 7.4. A log phase *E. coli* TG1 culture was infected with 0.75 ml of eluted phage and grown on TYE/A100/G2 bioassay plates at 30 °C overnight. The resulting colonies were scraped from the plates and grown to log phase in 2xTY/A100/G2 media prior to M13K07 infection. Subsequent rounds of selection and rescue were repeated as above. Enrichment of target-specific clones was evaluated via the binding of polyclonal and monoclonal phage supernatant to ELISA plates coated with antigen at 1 μg/ml and blocked with 5% MPBS. Phage binding was detected with anti-M13 phage coat G8p mAb at a 1:1000 dilution (Invitrogen, catalogue number MA1-06603) followed by anti-mouse HRP antibody at a 1:1000 dilution (Sigma, catalogue number A6782). Plasmid was prepared from individual clones identified as being positive for antigen binding and their VNAR inserts sequenced using the vector-specific primers pHEN Seq (5’-CTA TGC GGC CCC ATT CA-3’) and LMB3 (5’-CAG GAA ACA GCT ATG AC-3’).

### DNA plasmid construction and preparation

SARS-CoV-2 Spike- or RBD-ferritin constructs were derived from the Wuhan-Hu-1 strain genome sequence (GenBank MN9089473), including RBD subunit (residues 331–527), or S ectodomain (residues 12–1158). Constructs were modified to incorporate a N-terminal hexa-histidine tag (His) for purification of the RBD-Ferritin construct. The His-tagged SARS-CoV-2 RBD molecules were generated by amplifying the RBD domain from the RBD-Ferritin plasmid while encoding the 3’ His purification tag and subcloned into the CMVR vector. DNA encoding the SARS-CoV-2 stabilized S-2P gene was provided by Dr. Kizzmekia Corbett and Barney Graham (NIH) and a C-terminal His-tag and StrepTagII were added. S-2P used the native leader sequence, and RBD, RBD-ferritin and SpFN used a prolactin leader (PL) sequence.

To generate the ShAb-Fc fusion proteins, the VNAR genes was amplified from pHEN2 while adding a NotI site and a leader sequence to the 5’ end and adding a XbaI site to the 3’ end. NotI and XbaI were used to subclone the VNAR genes into a version of the CMVR vector that already had the human Fc gene encoded. The VNARs were inserted upstream of the Fc region. The ShAb constructs used for crystallization were amplified in a similar manner, but at the 3’ end, we encoded a RSV3C protease site and a His8 purification tag and a BamHI site, to be used with the upstream NotI site for subcloning into the CMVR vector. The ShAb01H02K combination was generated by introducing a “hole” (Y407T) mutation in Fc region of the ShAb01 construct and a “knob” (T366Y) mutation in the Fc of ShAb02 by site-directed mutagenesis. The constructs were then co-transfected to generate ShAb01H02K through heterologous pairing. In the BiShAb0201 construct, the 02 VNAR and a (GGGGS)_5_ linker were added to the N terminus of ShAb01. For the trimeric ShAbs, the VNAR was fused to the Foldon trimerization domain via a KESGSVSSEQLAQFRSGD linker.

Plasmid DNA generated by subcloning (restriction digest and ligation) was amplified in and isolated from *E. coli* Top10 cells. The constructs resulting from site-directed mutagenesis were amplified in and isolated from either *E. coli* Stbl3 or Top10 cells. Large-scale DNA isolation was performed using endo-free Maxiprep, Megaprep, or Gigaprep kits (Qiagen).

### Recombinant protein expression

All expression vectors were transiently transfected into Expi293F cells (Thermo Fisher Scientific) using Turbo293 Transfection Reagent (Speed Biosystems), with Ab booster (ABI scientific) added 18 h after transfection for ShAb expression. Cells were grown in polycarbonate baffled shaker flasks at 34 °C or 37 °C and 8% CO_2_ at 120 rpm. Cells were harvested 5–6 days post-transfection via centrifugation at 2862 × g for 30 min. Culture supernatants were filtered with a 0.22 µm filter and stored at 4 °C prior to purification.

### Immunogen and Spike domain purification

His-tagged proteins were purified using Ni-NTA affinity chromatography, while untagged proteins were purified with GNA lectin affinity chromatography. Briefly, 25 mL GNA-lectin resin (VectorLabs) was used to purify untagged SpFN protein from 1 L of expression supernatant. GNA resin was equilibrated with 10 column volumes (CV) of phosphate buffered saline (PBS) (pH 7.4) followed by supernatant loading at 4 °C. Unbound protein was removed by washing with 20 CV of PBS. Bound protein was eluted with 250 mM methyl-α-D mannopyranoside in PBS buffer (pH 7.4). His-tagged proteins were purified using 1 mL Ni-NTA resin (Thermo Scientific) per 1 L of expression supernatant. Ni-NTA resin was equilibrated with 5 CV of PBS followed by supernatant loading at room temperature. Unbound protein was removed by washing with 200 CV of PBS, followed by 50 CV 10 mM imidazole in PBS. Purification purity for all the proteins was assessed by SDS-PAGE. RBD proteins were dialyzed (10 K molecular weight cutoff) against PBS; immunogens and Spike proteins were further purified by size-exclusion chromatography using a 16/60 Superdex-200 purification column. Removal of the His-tags for SARS2-CoV-2 S-2P and RBD for use in ELISA was achieved using HRV-3C protease. Endotoxin levels for ferritin nanoparticle immunogens were evaluated and 5% v/v glycerol was added prior to filter-sterilization with a 0.22 µm filter, flash-freezing in liquid nitrogen, and storage at −80 °C. Ferritin nanoparticle formation was assessed by Dynamic light scattering by determining the hydrodynamic diameter at 25 °C using a Malvern Zetasizer Nano S (Malvern, Worcestershire, UK) equipped with a 633 nm laser.

### ShAb and mAb antibody purification

ShAbs or mAbs were purified with Protein A affinity chromatography. rProtein A Sepharose™ Fast Flow Affinity Media (GE Healthcare/Cytiva) from 1 L of expression supernatant. Protein A resin was equilibrated with 20 CV of PBS followed by supernatant loading at room temperature. Unbound protein was removed by washing with 60 CV of PBS. Bound protein was eluted with IgG Elution Buffer (Thermo Scientific) and neutralized with 0.1 M Tris pH 8.0. Purification purity was assessed by SDS-PAGE under reducing and nonreducing conditions. For long-term storage, Fc-fusion antibodies were filter-sterilized with a 0.22-µm filter, flash-frozen in liquid nitrogen, and stored at −80 °C.

### X-ray crystallography

The SARS-CoV-2 RBD-ShAb01 VNAR-ShAb02 VNAR complex (9.5 mg/ml) were screened for crystallization conditions using an Art Robbins Gryphon crystallization robot, 0.2 µl drops, and a set of 1200 conditions by hanging-drop vapor diffusion at 293 K. Crystal drops were observed daily using a Jan Scientific UVEX-PS hotel with automated UV and brightfield drop imaging. Initial crystallization conditions were optimized manually in larger 1 µl drops, and crystals used for data collection grew in the following crystallization conditions: 0.1 M HEPES pH7.4, 14.5% PEG20000.

Single crystals were transferred to mother liquor containing 20–25% glycerol, and cryo-cooled in liquid nitrogen prior to data collection. Diffraction data were collected at APS 24-ID-E beamline using RAPD, a modular package of programs written for the automated processing of macromolecular crystallographic data. Data was measured using a Dectris Eiger 16 M PIXEL detector to a final resolution of 2.5 Å. Final diffraction data indexing, integration, and scaling were carried out using the HKL2000 suite^27^. Data collection statistics are reported in Supplementary Table 3.

Phenix.xtriage was used to analyze all the scaled diffraction data output from HKL2000 and XDS. Primarily, data was analyzed for measurement value significance, completeness, asymmetric unit volume, and possible twinning and/or pseudotranslational pathologies. The crystal structure described in this study was solved by molecular replacement using the program Phaser. Refinement for the structure model was carried out using Phenix refine with positional, global isotropic B-factor refinement and defined TLS groups. Manual model building was performed in Coot 0.8.9.2. All structure figures were generated using PyMOL 2.3.2 (The PyMOL Molecular Graphics System, Schrodinger).

### Octet biolayer interferometry binding and ACE2 inhibition assays

All biosensors were hydrated in PBS prior to use. All assay steps were performed at 30 °C with agitation set at 1000 rpm in the Octet RED96 instrument (fortéBio).

Biosensors were equilibrated in assay buffer (PBS) for 30 s before loading of IgG antibodies (30 µg/ml diluted in PBS). ShAbs were immobilized onto AHC biosensors (fortéBio) for 100 s, followed by a brief baseline in assay buffer for 15 s. Immobilized antibodies were then dipped in various antigens for 100–180 s followed by dissociation for 100–180 s. Affinity kinetic constants between SARS-CoV-2 RBD molecules and ShAbs were determined, using at least 4 concentrations of RBD, by fitting the curves to a 1:1 Langmuir binding model using the Data analysis software 9.0 (ForteBio).

ShAbs were assessed for the ability to block ACE2 binding to SARS CoV-2 S-2P, RBD and a set of sarbecovirus RBD molecules. ACE2-inhibition assays were carried out as follows. RBD or S-2P (30 μg/ml diluted in PBS) was immobilized on HIS1K biosensors (fortéBio) for 180 s followed by baseline equilibration for 30 s. Probes were then incubated with different concentrations of a given ShAb and binding was allowed to occur for 180 s followed by baseline equilibration (30 s). ACE2 protein (30 μg/ml) was then allowed to bind for 120 s. Percent inhibition (PI) of RBD binding to ACE2 by ShAb-Fc were determined using the equation: PI = 100 − ((ACE2 binding in the presence of ShAb-Fc) ⁄ (ACE2 binding in the absence of ShAb-Fc)) × 100).

### Enzyme linked immunosorbent assay (ELISA)

96-well Immulon “U” Bottom plates were coated with 1 μg/mL of RBD or spike protein (S-2P) antigen in 0.1 M sodium bicarbonate. Plates were incubated at 4 °C overnight and blocked with blocking buffer (Dulbecco’s PBS containing 0.2% bovine serum albumin, pH 7.4), at room temperature (RT) for 30 min. Protein samples were serially diluted 5-fold in sample buffer (Dulbecco’s PBS containing 0.2% bovine serum albumin and 0.05% Tween 20, pH 7.4), added to duplicate wells and the plates were incubated for 1 h at RT. Horseradish peroxidase (HRP)-conjugated goat anti-human IgG, gamma chain specific antibody (Sigma, catalogue number A8419-2ML) was added at a 1:10,000 dilution and incubated at RT for 30 min. After each step, the plates were washed 3–4 times with wash buffer (Dulbecco’s PBS containing 0.05% Tween 20, pH 7.4). For color development, the substrate mixture from TMB Substrate set (Biolegend) was added and incubated for 16 min, before the addition of the Stop Solution for TMB Substrate (Biolegend). Absorbance (A) was measured at 450 nm using an ELISA reader Spectramax (Molecular Devices, San Jose, CA).

### SARS-CoV-2, SARS-CoV-2 VoC and SARS-CoV-1 pseudovirus neutralization assay

The S expression plasmid sequences for SARS-CoV-2 and SARS-CoV-1 were codon optimized and modified to remove an 18 amino acid endoplasmic reticulum retention signal in the cytoplasmic tail in the case of SARS-CoV-2, and a 28 amino acid deletion in the cytoplasmic tail in the case of SARS-CoV-1. This allowed increased S incorporation into pseudovirions (PSV) and thereby improved infectivity. Virions pseudotyped with the vesicular stomatitis virus (VSV) G protein were used as a non-specific control. SARS-CoV-2 pseudovirions (PSV) were produced by co-transfection of HEK293T/17 cells with a SARS-CoV-2 S plasmid (pcDNA3.4) and an HIV-1 NL4-3 deltaEnv luciferase reporter plasmid (pNL4-3.Luc.R-E-, NIH AIDS Reagent Program). The SARS-CoV-2 S expression plasmid sequence was derived from the Wuhan seafood market pneumonia virus isolate Wuhan-Hu-1, complete genome (GenBank accession MN908947), and the SARS-CoV-1 expression plasmid was derived from the Urbani S sequence. S expression plasmids for current SARS-CoV-2 VoC were similarly codon optimized, modified and included the following mutations: B.1.1.7/Alpha, (69-70del, Y144del, N501Y, A570D, D614G, P681H, T718I, S982A, D1118H), B.1.351/Beta, (L18F, D80A, D215G, 241-243del, K417N, E484K, N501Y, D614G, A701V, E1195Q), B.1.617.2/Delta, (T19R, G142D, del156-157, R158G, L452R, T478K, D614G, P681R, D950N), B.1.529/Omicron BA.1 (A67V, del69/70, T95I, G142D, ΔV143-Y145, delN211, L212I, ins214EPE, G339D, S371L, S373P, S375F, K417N, N440K, G446S, S477N, T478K, E484A, Q493R, Q498R, N501Y, Y505H, T547K, D614G, H655Y, N679K, P681H, N764K, D796Y, N856K, Q954H, N969K, L981F), and B.1.529/Omicron BA.4/5 (T19I, L24S, del25/27, del69/70, G142D, V213G, G339D, S371F, S373P, S375F, T376A, D405N, R408S, K417N, N440K, L452R, S477N, T478K, E484A, F486V, Q498R, N501Y, Y505H, D614G, H655Y, N679K, P681H, N764K, D796Y, Q954H, N969K).

Infectivity and neutralization titers were determined using ACE2-expressing HEK293 target cells (Integral Molecular) in a semi-automated assay format using robotic liquid handling (Biomek NXp Beckman Coulter). Antibodies were diluted in growth medium and serially diluted, then 25 µL/well was added to a white 96-well plate. An equal volume of diluted SARS-CoV-2 PSV was added to each well and plates were incubated for 1 h at 37 °C. Target cells were added to each well (40,000 cells/ well) and plates were incubated for an additional 48 h. RLUs were measured with the EnVision Multimode Plate Reader (Perkin Elmer, Waltham, MA) using the Bright-Glo Luciferase Assay System (Promega Corporation, Madison, WI). Neutralization dose–response curves were fitted by nonlinear regression using the LabKey Server®, and the final titers are reported as the reciprocal of the dilution of serum necessary to achieve 50% neutralization (ID50, 50% inhibitory dilution). The VNAR-ShAb comparisons shown in Supplementary Fig. 9 were independent experiments to those shown in Fig. 6. Assay equivalency for SARS-CoV-2 was established by participation in the SARS-CoV-2 Neutralizing Assay Concordance Survey (SNACS) run by the Virology Quality Assurance Program and External Quality Assurance Program Oversite Laboratory (EQAPOL) at the Duke Human Vaccine Institute, sponsored through programs supported by the National Institute of Allergy and Infectious Diseases, Division of AIDS.

### K18-hACE2 transgenic mouse passive immunization and challenge

All research in this study involving animals was conducted in compliance with the Animal Welfare Act, and other federal statutes and regulations relating to animals and experiments involving animals and adhered to the principles stated in the Guide for the Care and Use of Laboratory Animals, NRC Publication, 1996 edition. The research protocol was approved by the Institutional Animal Care and Use Committee of the Trudeau Institute, protocol 20–007. K18-hACE2 transgenic mice were obtained from Jackson Laboratories (Bar Harbor, ME). Mice were housed in the animal facility of the Trudeau Institute and cared for in accordance with local, state, federal, and institutional policies in a National Institutes of Health American Association for Accreditation of Laboratory Animal Care-accredited facility. Animals were maintained in IVC cages on negatively pressurized Allentown PNC racks that were HEPA filtered and directly vented though the building’s exhaust system. The racks and animal rooms were negatively pressurized. Access to all facilities is controlled electronically by the buildings management systems and restricted to approved users only. The environment, temperature, and humidity, within the animal facility room were constantly monitored by the building management system. Temperatures were also monitored and recorded daily in individual animal rooms by animal care staff using electronic thermometers. All temperature set points were within The Guide recommended range of 68–79 °F for mice. Acceptable Institutional daily fluctuations are between 67 and 74 °F with a humidity range of 30–70%. Light cycles in all animal holding and procedure spaces are controlled on a 12/12 light/dark cycle.

For the passive immunization studies, one day prior to challenge, 200 μg of ShAb01, ShAb02, or an isotype control were injected into the intraperitoneal cavity of three groups of K18-hACE2 mice. Each group consisted of 13 mice (7 female, 6 male), aged 6–10 weeks old. On study day 0, all mice were infected with 1.25 × 10^4^ PFU of SARS-CoV-2 USA-WA1/2020 via intranasal instillation. The viral titer used for infection was previously determined to provide robust and reproducible infection, and consistent weight loss in this model. The day 2 timepoint for recovery and analysis of lung viral load was determined as the timepoint with peak lung viral loads using this challenge virus stock. All mice were monitored for clinical symptoms and body weight twice daily, every 12 h, from study day 0 to study day 14. Mice were euthanized if they displayed any signs of pain or distress as indicated by the failure to move after stimulation or presentation of inappetence, or if a mouse had > 25% weight loss compared to their study day 0 body weight. Animals were assigned a clinical score as follows: 0: normal appearance and movement, 1: slightly ruffled fur, 2: slightly ruffled fur and reduced mobility, 3: slightly ruffled fur, reduced mobility, and rapid breathing, 4: slightly ruffled fur, reduced mobility, rapid breathing and hunched and huddled stance, and 5: found dead or euthanized due to weight loss or being moribund. K18-hACE2 mouse survival comparisons were carried out using GraphPad using Gehan-Breslow-Wilcoxon test. Two days post-challenge, viral loads were measured by plaque assay in the bronchoalveolar lavage of 5 animals from each group.

### Weighing epitope sites based on antigen-antibody interactions

Epitope sites correspond to antigen sites that are in contact with the antibody in the antigen-antibody complex (i.e., all sites that have non-hydrogen atoms within 4 Å of the antibody). For a given epitope site, the weight, which characterizes the interaction between the epitope site and the antibody (improved based on), was defined as:

1 $$w=\frac{1}{2}\left(\frac{{n}_{c}}{\left\langle {n}_{c}\right\rangle }+\frac{{n}_{{nb}}}{\left\langle {n}_{{nb}}\right\rangle }\right)$$

in which, *n*_*c*_ is the number of contacts with the antibody (i.e. the number of non-hydrogen antibody atoms within 4 Å of the site); *n*_*nb*_ is the number of neighboring antibody residues; 〈*n*_*c*_〉 is the mean number of contacts *n*_*c*_ and 〈*n*_*nb*_〉 is the mean number of neighboring antibody residues *n*_*nb*_ across all epitope sites. A weight of 1.0 is attributed to the average interaction across all epitope sites. Neighboring residue pairs were identified by Delaunay tetrahedralization of side-chain centers of residues (C_a_ is counted as a side chain atom, pairs further than 8.5 Å were excluded). Quickhull was used for the tetrahedralization and Biopython PDB to handle the protein structure. In the SARS-CoV-2 and SARS-CoV-1 RBD comparison, residues were considered similar for the following residues pairs: RK, RQ, KQ, QE, QN, ED, DN, TS, SA, VI, IL, LM, and FY.

### Epitope mapping of antibodies by alanine scanning

Epitope mapping was performed essentially as previously reported (Davidson et al., 2014) using SARS-CoV-2 (strain Wuhan-Hu-1) RBD. 184 residues of the RBD (S residues 335–526) were mutated individually to alanine, and alanine residues to serine. Mutations were confirmed by DNA sequencing, and clones arrayed in 384-well plates, one mutant per well. Binding of mAbs to each mutant clone in the alanine scanning library was determined, in duplicate, by high-throughput flow cytometry. Each S protein mutant was transfected into HEK-293T cells and allowed to express for 22 h. Cells were fixed in 4% (v/v) paraformaldehyde (Electron Microscopy Sciences), and permeabilized with 0.1% (w/v) saponin (Sigma-Aldrich) in PBS plus calcium and magnesium (PBS++) before incubation with mAbs diluted in PBS++, 10% normal goat serum (Sigma), and 0.1% saponin. mAb screening concentrations were determined using an independent immunofluorescence titration curve against cells expressing wild-type S protein to ensure that signals were within the linear range of detection. mAbs were detected using 3.75 μg mL^−1^ of AlexaFluor488-conjugated secondary antibody (Jackson ImmunoResearch Laboratories) in 10% normal goat serum with 0.1% saponin. Cells were washed three times with PBS++/0.1% saponin followed by two washes in PBS and mean cellular fluorescence was detected using a high-throughput Intellicyte iQue flow cytometer (Sartorius). mAb reactivity against each mutant S protein clone was calculated relative to wild-type S protein reactivity by subtracting the signal from mock-transfected controls and normalizing to the signal from wild-type S-transfected controls. Mutations within clones were identified as critical to the mAb epitope if they did not support reactivity to the test mAb but supported reactivity of other SARS-CoV-2 antibodies. This counter-screen strategy facilitates the exclusion of S mutants that are locally misfolded or have an expression defect.

### Antibody-dependent cellular phagocytosis

ADCP was measured as previously described^42^. Briefly, SARS-CoV-1 and SARS-CoV-2 S proteins were biotinylated according to manufacturer’s instructions (ThermoFisher Scientific), at a biotin to protein ratio of 50, and then incubated with yellow-green streptavidin-fluorescent beads (Molecular Probes) for 2 h at 37 °C. 10 ul of a 100-fold dilution of beads-protein mixture was incubated for 2 h at 37 °C with 100ul of VNAR (1 ug/ml, 0.2 ug/ml, and 0.04 ug/ml), before addition of THP-1 cells (25,000 cells per well; Millipore Sigma, Burlington, MA, USA). After 19 h incubation at 37 °C, the cells were fixed with 4% formaldehyde solution (Tousimis, Rockville, MD, USA) and fluorescence was evaluated on a LSRII (BD Biosciences). The phagocytic score was calculated by multiplying the percentage of bead-positive cells by the geometric mean fluorescence intensity (MFI) of the bead-positive cells and dividing by 10,000.

### NK cell activation assay

PBMC from one control were rested overnight with 10 ng/ml human recombinant IL-15 (R&D Sytems) and NK cells were then isolated using EasySep Human NK Cell Isolation Kit (Stemcell Technologies). Nunc MaxiSorp Flat-Bottom plates (ThermoFisher) were coated with 1.5 ng/well of SARS-CoV-1 or SARS-CoV-2 spike proteins and incubated at 4 C for 6 h, then washed and blocked with R10 for 19 h at 4 C. After blocking, 100 ul of vNARs (5ug/ml) were added to each well and plates were incubated 30 min at 37 C. Plates were then washed with PBS 4x, followed by addition of 25,000 purified NK cells per well. GolgiStop (BD Biosciences) and Brefeldin A (ThermoFisher) were also added at 1:1000, and CD107a APC (clone H4A3; BD Biosciences) was added 1:100 in R10. After 6 h at 37 C, cells were stained for surface markers Aqua Live/Dead stain (ThermoFisher), CD3 PE-Tx Red (clone 7D6; ThermoFisher), CD56 PE-Cy7 (clone NCAM16.2; BD Biosciences), CD19 AF700 (clone HIB19; BD Biosciences), and CD16 BUV496 (clone 3G8; BD Biosciences) in FACS Buffer for 10 min at RT. Cells were fixed with Fix & Perm Medium A (ThermoFisher) for 15 m at RT. Fluorescence was evaluated on a LSRII (BD Biosciences). Data was analyzed using FlowJo version 10.7.1. NK cells were gated as CD3-CD19-/CD56+ CD16+, and CD107a expression was evaluated.

### Reporting summary

Further information on research design is available in the Nature Portfolio Reporting Summary linked to this article.

## Supplementary Information

Supplementary Information

Reporting Summary

## Data Availability

The atomic model is deposited in the PDB under accession number PDB 7S83. All other data are available in the main manuscript, Supplementary Information, or the Source Data file provided with this paper. The ShAb molecules described in this study will be made available to the scientific community by contacting M.G.J. and upon completion of a materials transfer agreement. Source data are provided with this paper.

## Source data

Source Data
