# Supplementary material for: Shark nanobodies with potent SARS-CoV-2 neutralizing activity and broad sarbecovirus reactivity
Source: Nat Commun. 2023 Feb 3;14:580. doi: 10.1038/s41467-023-36106-x (PMC9896449; doi:10.1038/s41467-023-36106-x)
Supplement: Supplementary file 1 — Supplementary Information [file 41467_2023_36106_MOESM1_ESM.pdf]

Supplementary Information

Shark nanobodies with potent SARS-CoV-2 neutralizing activity and broad sarbecovirus reactivity

Wei-Hung Chen[1,2,9], Agnes Hajduczki[1,2,9], Elizabeth J. Martinez[1,2], Hongjun Bai[1,2,3], Hanover Matz[4], Thomas M. Hill[4], Eric Lewitus[1,2,3], William C. Chang[1,2], Layla Dawit[1,2], Caroline E. Peterson[1,2], Phyllis A. Rees[1,2], Adelola B. Ajayi [1,2], Emily S. Golub[1,2], Isabella Swafford[1,2,3], Vincent Dussupt[1,2,3], Sapna David[1,2], Sandra V. Mayer[2,5], Sandrine Soman[5], Caitlin Kuklis[5], Courtney Corbitt[1,2,5], Jocelyn King[1,2,5], Misook Choe[1,2], Rajeshwer S. Sankhala[1,2], Paul V. Thomas[1,2], Michelle Zemil[3], Lindsay Wieczorek[2,3], Tricia Hart[6], Debora Duso[6], Larry Kummer[6], Lianying Yan[7], Spencer L. Sterling[7], Eric D. Laing[7], Christopher C. Broder[7], Jazmean K. Williams[8], Edgar Davidson[8], Benjamin J. Doranz[8], Shelly J. Krebs[1,2,3], Victoria R. Polonis[3], Dominic Paquin-Proulx[1,2,3], Morgane Rolland[1,2,3], William Reiley[6], Gregory D. Gromowski[5], Kayvon Modjarrad[1], Helen Dooley[4,*], and M. Gordon Joyce[1,2,6,*]

1. Emerging Infectious Diseases Branch, Walter Reed Army Institute of Research, Silver Spring, MD, USA.
2. Henry M. Jackson Foundation for the Advancement of Military Medicine, Bethesda, MD, USA.
3. U.S. Military HIV Research Program, Walter Reed Army Institute of Research, Silver Spring, MD, USA.
4. Department of Microbiology and Immunology, University of Maryland School of Medicine, Baltimore, MD, USA; Institute of Marine and Environmental Technology, Baltimore, MD, USA.
5. Viral Diseases Branch, Walter Reed Army Institute of Research, Silver Spring, MD, USA.
6. Trudeau Institute, Saranac Lake, NY, USA.
7. Department of Microbiology and Immunology, Uniformed Services University, Bethesda, MD, USA, Bethesda, MD, USA.
8. Integral Molecular, Philadelphia, PA, USA.
9. These authors contributed equally.

*Correspondence: hdooley@som.umaryland.edu (H.D.), and gjoyce@eidresearch.org (M.G.J)

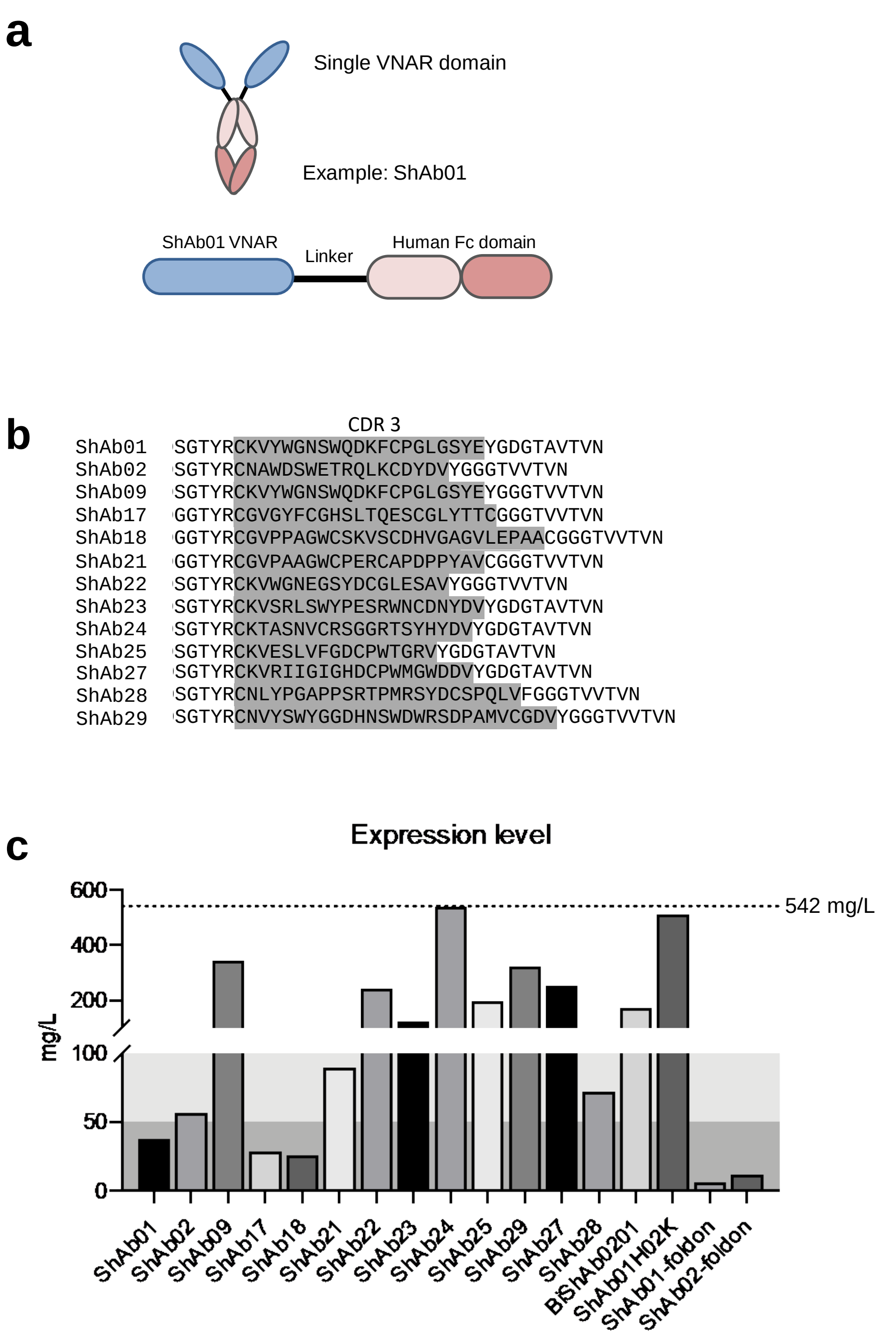

Supplementary Fig. 1. Fc chimera schematic, VNAR ShAb CDR3 sequences and expression level of ShAb molecules.
a, Schematic of the VNAR-Fc nanobody design.
b, Sequence alignment of the CDR3 regions of the ShAb VNARs.
c, ShAb protein expression levels in mammalian Expi293F cells by transient transfection.
Source data are provided as a Source Data file.

Supplementary Fig. 2.

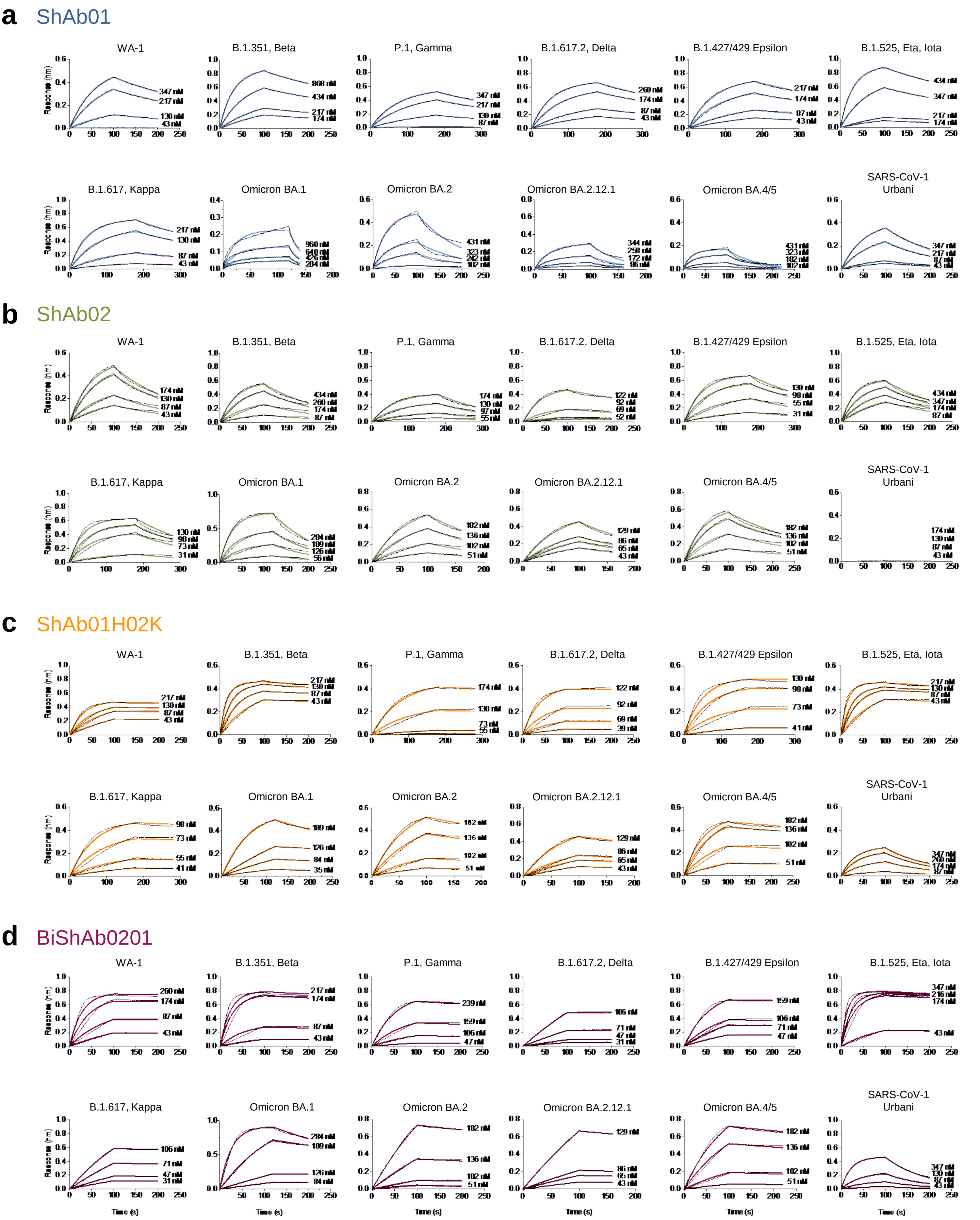

Supplementary Fig. 2. ShAb affinity to SARS-CoV-2 VoC and SARS-CoV-1 RBDs. BLI binding kinetic measurements were carried out using immobilized ShAb molecules, and the RBD molecules in solution. Representative binding curves (black lines) are fitted to a 1:1 binding model (colored lines).
Source data are provided as a Source Data file.

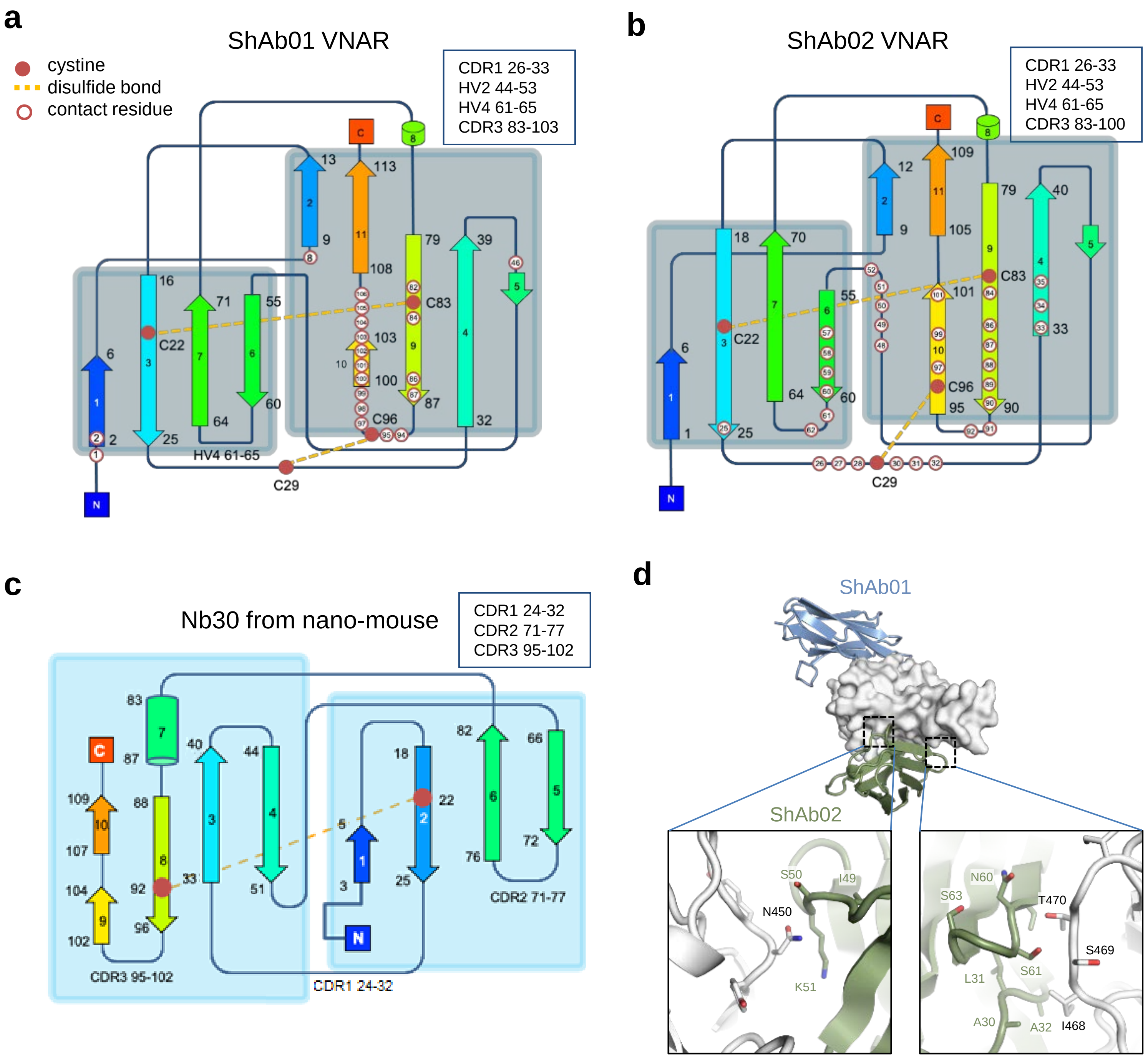

e

```
       1                      25                       50                       75                      100
ShAb01 ARVDQTPRSVTKETGESLTINCVLRDSNCALSSTHWYRKKSGSTNEERILQGRRYVETVNSGSKSFSLRINDLRVEDSGTYRCKVYWGNSWQDKFCPGLGSYEYGDGTAVTVN
       ☼☼     ☼                                     ☼                                   ☼☼☼ ☼☼.     ○●○●○○○●●●●○☼
ShAb02 ARVDQTPQTITKETGESLTINCVLRDSNCALASTDWYRKKSGSTNEESISKGGRYVETVNSGSKSFSLRINDLTVEDSGTYRCNAWDSWETRQLKCDYDV---YGGTVVTVN
                                   ☼☼○☼○○☼            ☼○●●○      ●○●○                     ☼ ☼☼○●☼○☼    ☼ ☼    ☼

○ main chain interaction
● main chain and side chain interaction
☼ side chain interaction
```

Supplementary Fig. 3. Structural analysis of ShAb01 and ShAb02 VNARs.
a, b, c, Topology diagram of ShAb01, ShAb02, and Nb30 with β-strands (arrows) and α-helices (tube) drawn and labeled. Cysteines forming disulfide bonds are shown as solid red circles, disulfide bonds are shown as yellow dashed lines. SARS-CoV-2 RBD contacting residues are shown as open circles with numbers.
d, Contact residues of ShAb02 in complex with SARS-CoV-2 RBD.
e, ShAb01, and ShAb02 VNAR sequence with paratope contact residues indicated (open sphere: main chain-only contact; closed sphere: main- and side-chain contacts; sun sphere: side-chain-only contacts).

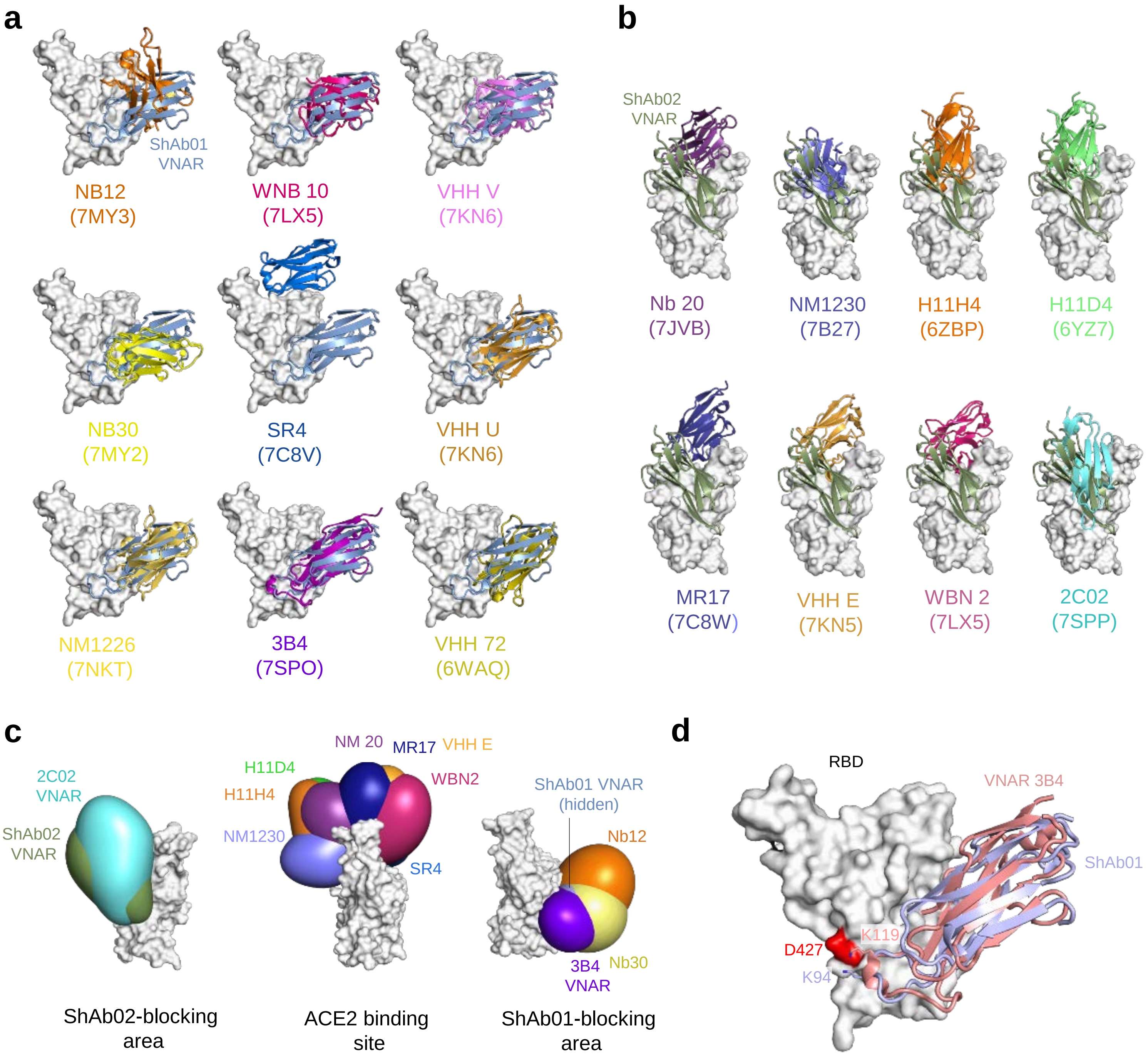

e

```
Shab01  ARVDQTPRSVTKETGESLTINCVLRDSNCALSSTHWYRKKSGSTNEERILQGRRYVETVN 60
VNAR3B4 ASVNQTPRTATKETGESLTINCVVTGARCGLSRTSWFRKNPGTTDWERMSIGGRYVESVN 60
        * *:****:.*************: .:.*.** * *:**: *:*: **:  * ****:**

Shab01  SGSKSFSLRINDLRVEDSGTYRCKVYWGNS------WQDKFCPGLGSYEYGDGTAVTVNG 114
VNAR3B4 KGAKSFSLRIKDLTVADSATYICRAWSDTSQKPCHAWEQKMWEGH---VDGAGTVLTVNQ 117
        .*:*******:** * **.** *:.: ..*      *::*:  *.     * **.:***
```

Supplementary Fig. 4. ShAb01 and ShAb02 VNAR RBD epitope comparison.
a, Structural overlay of ShAb01 VNAR in complex with SARS-CoV-2 RBD with previously described nanobodies (PDB IDs are shown).
b, Structural overlay of ShAb02 VNAR in complex with SARS-CoV-2 RBD with previously described nanobodies.
c, SARS-CoV-2 RBD (gray) is shown in surface representation, with ShAb01 and ShAb02 and other nanobodies shown in smooth surface representation.
d, Structure overlays of ShAb01 and VNAR 3B4 (PDB:7SPO), VNAR 3B4 contact residue K119 and ShAb01 K94 which form a salt bridge with D427 are shown in stick representation.
e, Sequence alignment of VNAR 3B4 and ShAb01 VNAR domain sequences.

Supplementary Fig. 5.

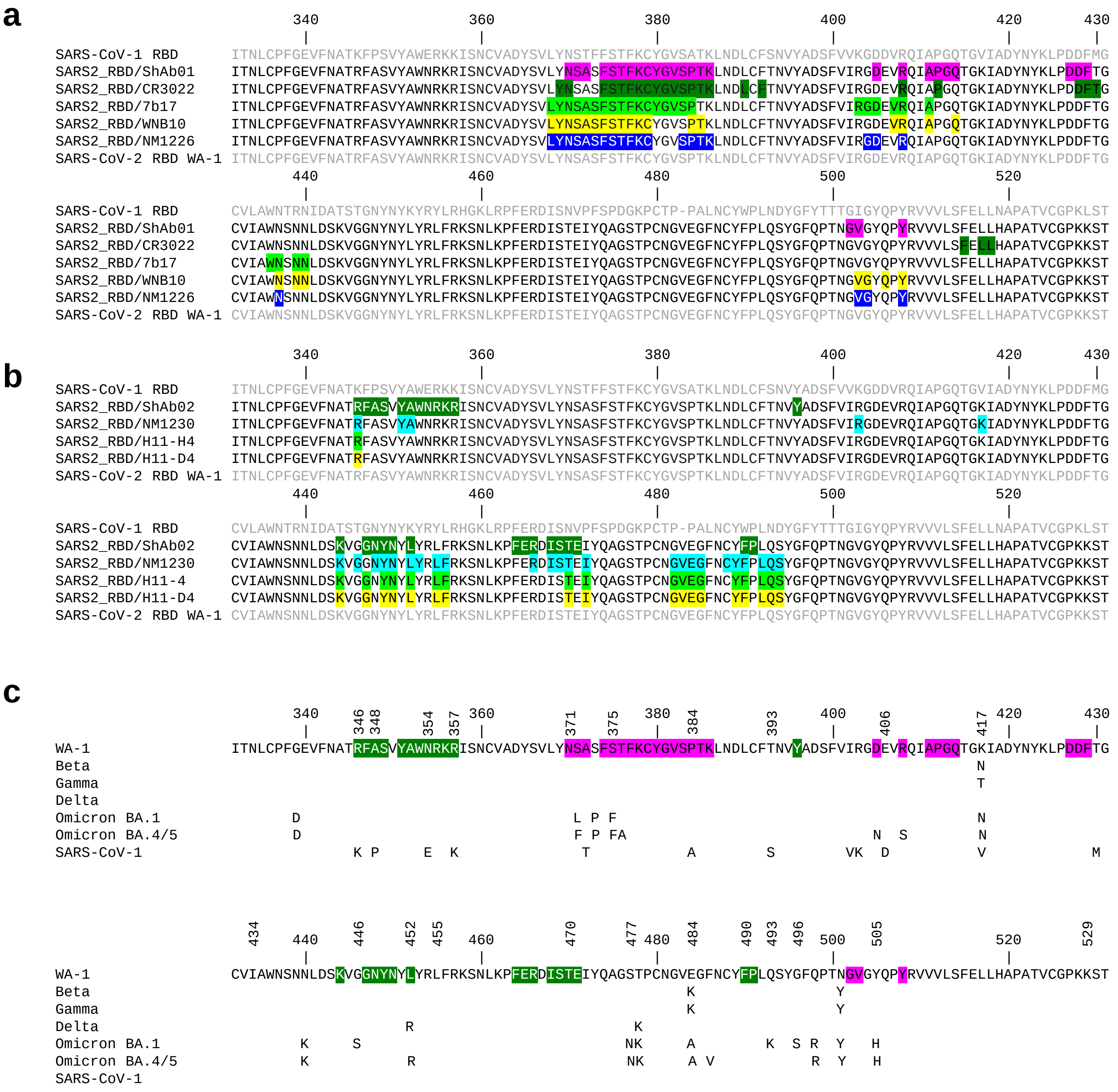

Supplementary Fig. 5. Sequence alignment of RBD antibody and nanobody epitopes.
a, Sequence alignment of RBD molecules with epitope residues highlighted for ShAb01 (colored green), CR3022 and other nanobodies with overlapping epitopes.
b, Sequence alignment of RBD molecules with epitope residues highlighted for ShAb02 (colored purple), and nanobodies with overlapping epitopes.
c, Sequences alignment of RBD molecule (residues N331 - S530) of VoCs. Mutations are indicated using the 1-letter amino acid convention.

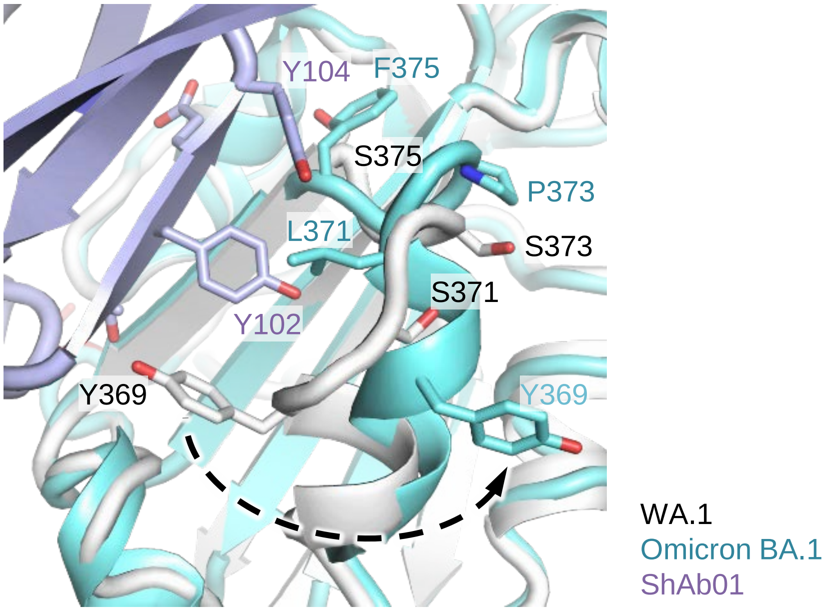

Supplementary Fig. 6. Comparison of the ShAb01 (purple) recognition in the context of the WA-1 RBD (white) and Omicron BA.1 (cyan). The Omicron BA.1 RBD model is taken from PBD:7WPB with all molecules shown in ribbon representation.

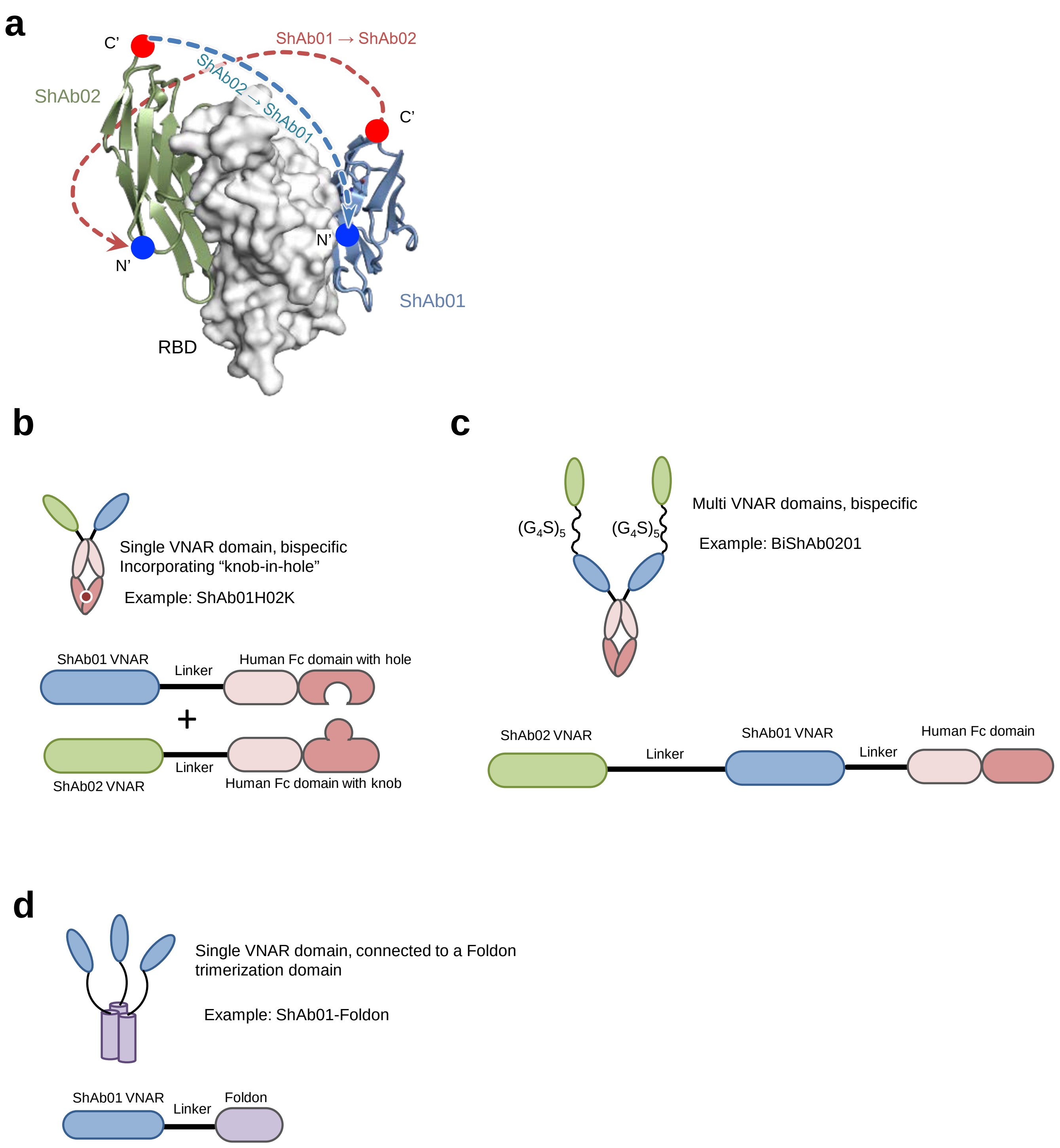

Supplementary Fig. 7. Construction of multi-domain ShAb nanobodies.
a, SARS-CoV-2 RBD is shown in surface representation with ShAb01 and ShAb02 VNARs in ribbon representation. The linkage of ShAb02 to ShAb01, or ShAb01 to ShAb02 are indicated by dashed lines.
b, Single VNAR domain, bispecific nanobody Incorporating "knob-in-hole" e.g., ShAb01H02K. c, multi-VNAR on a single chain, bi-specific, e.g., BiShAb0201.
d, single VNAR connected to a Foldon trimerization domain, e.g., ShAb01-Foldon.

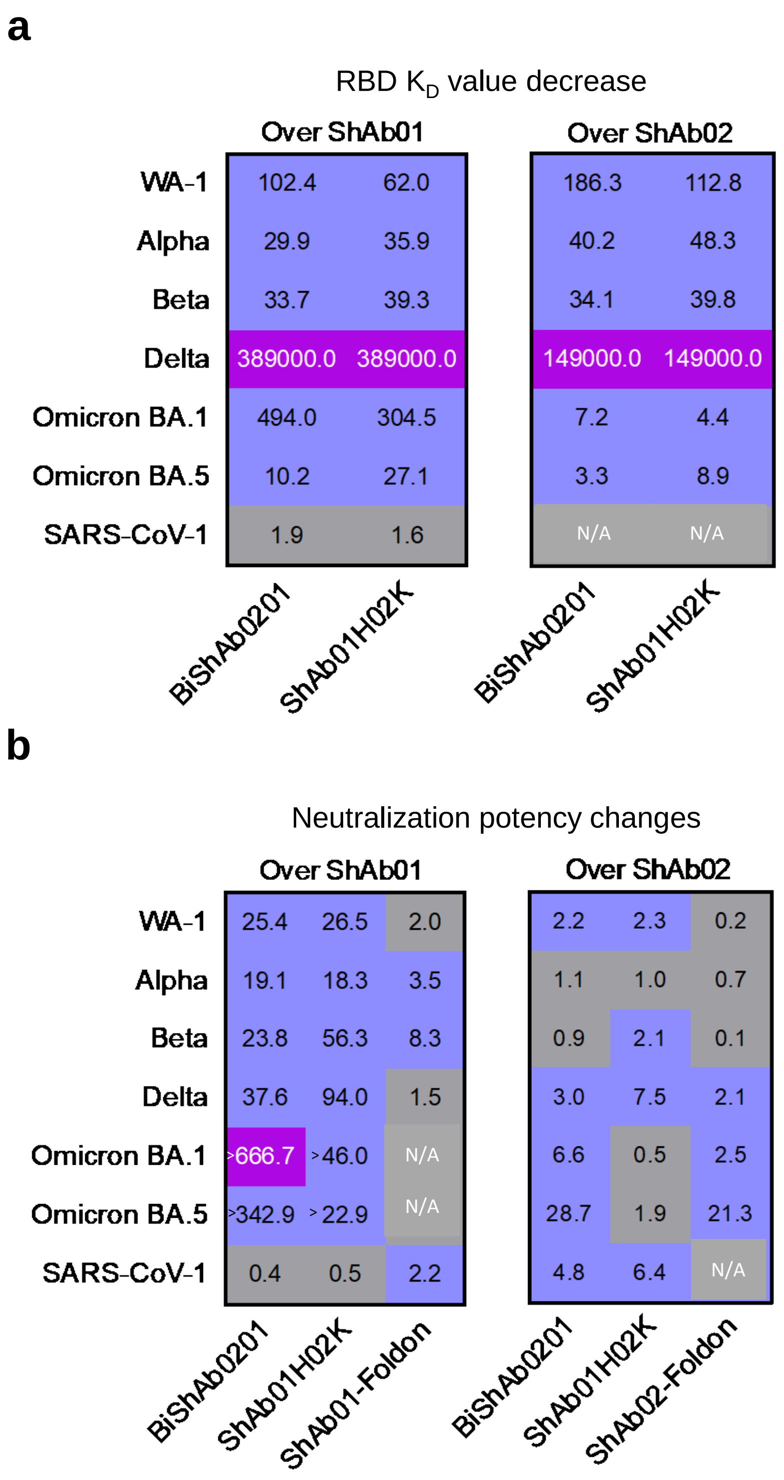

Supplementary Fig. 8. Change in binding and pseudovirus neutralization levels for multi-specific nanobodies relative to parental ShAb molecules.
a, Fold increase in RBD binding affinity of multi-specific ShAbs compared to ShAb01 and ShAb02.
b, Fold change in pseudovirus neutralization IC50 potency of multi-domain ShAbs compared to ShAb01 and ShAb02.
Source data are provided as a Source Data file.

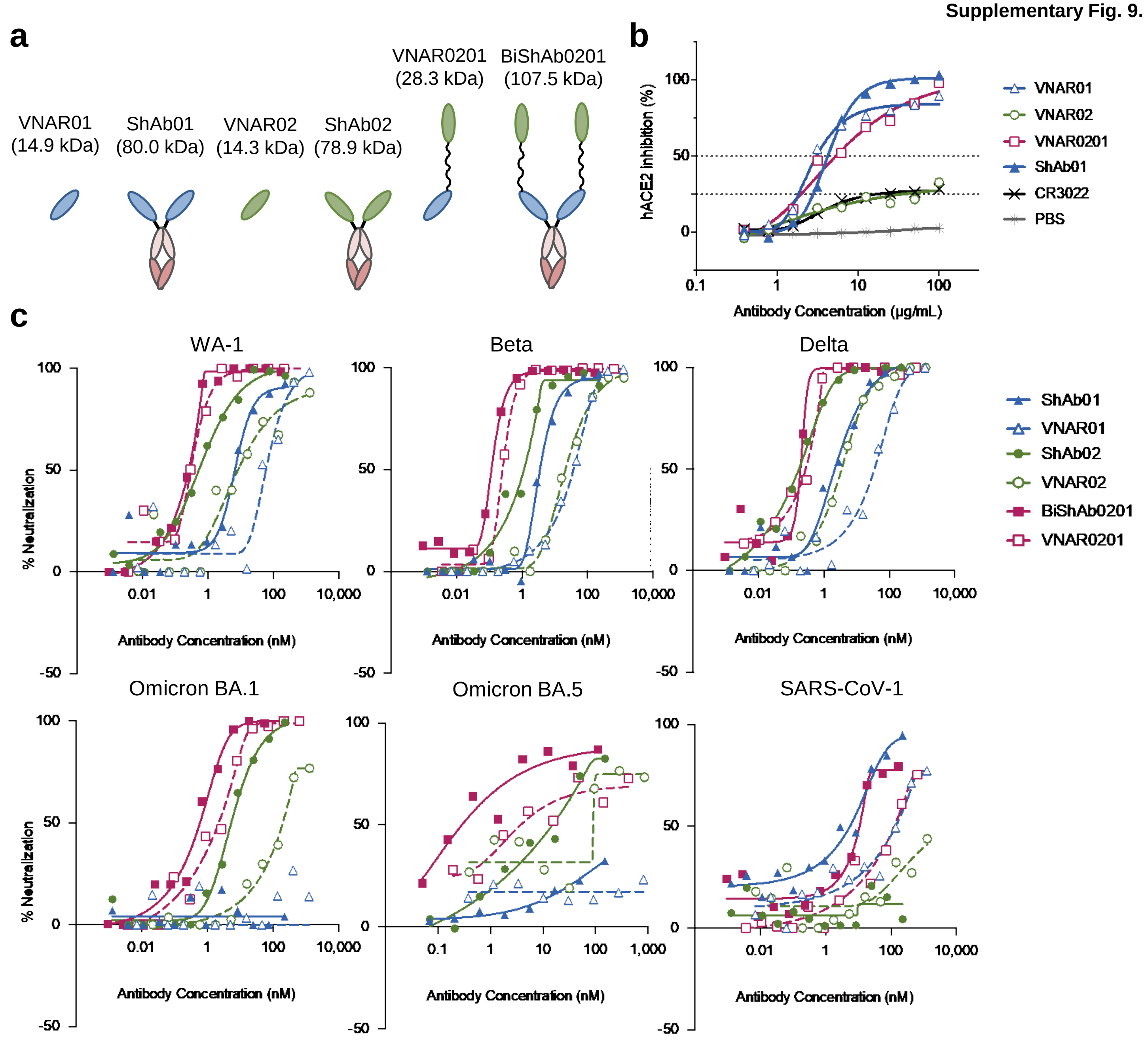

d

	VNAR01	ShAb01	VNAR02	ShAb02	VNAR 0201	BiShAb 0201
MW	14.6	80	14	78.9	28.3	107.5
	IC$_{50}$ (ng/ml)					
WA-1	907	535	134	42	10	26
Beta	502	295	292	63	7	14
Delta	517	198	45	13	6	18
Omicron BA.1	>18,000	>18,000	2,179	430	59	61
Omicron BA.4/5	>12,000	>12,000	631	1,003	165	35
SARS-CoV-1	2,026	298	>18,000	>18,000	3,074	1,211
	IC$_{50}$ (nM)					
WA-1	62.29	6.69	9.57	0.53	0.35	0.24
Beta	34.48	3.69	20.84	0.80	0.25	0.13
Delta	35.51	2.48	3.21	0.17	0.21	0.18
Omicron BA.1	n/a	n/a	155.53	5.45	2.09	0.57
Omicron BA.4/5	n/a	n/a	45.04	12.71	5.83	0.33
SARS-CoV-1	139.15	3.73	n/a	n/a	108.62	11.27

Supplementary Fig. 9. Evaluation of ShAbs compared to their VNAR-only counterparts.
a, Schematic representation of ShAbs and VNARs.
b, BLI measurement of ACE2-inhibition to SARS-CoV-2 WA-1 RBD. The measurements are performed with immobilized SARS-CoV-1 RBD, with ShAb molecules and hACE2 in solution.
c, Neutralization of SARS-CoV-2 WA-1, Beta, Delta, Omicron BA.1, Omicron BA.4/5, and SARS-CoV-1 Urbani pseudoviruses.
d, Neutralization table to compare the IC$_{50}$ of VNAR0201 and BiShAb0201. ShAb and VNAR IC$_{50}$ values for neutralization of each pseudovirus, are shown in ng ml^{-1} and nanomolar (nM).
Source data are provided as a Source Data file.

Supplementary Table 1. Immunization schedule and ShAb identification

Immunogen	Shark	Gender	Week of collection for library construction	Panning target	ShAb
RBD protein (RBD)	‘Pink’	female	15	RBD	01
RBD protein (RBD)	‘Red’	female	10	RBD	02
RBD-Ferritin (RFN)	‘Green’	male	38+40	SpFN	17-18
RBD-Ferritin (RFN)	‘Yellow’	female	38+40	SpFN	21
Spike-Ferritin (SpFN)	‘Purple’	female	10	SpFN	09
Spike-Ferritin (SpFN)	‘Purple’	female	10	RFN	28-29
Spike-Ferritin (SpFN)	‘Blue’	male	10	RFN	22-27

Supplementary Table 2. Binding kinetics of ShAb01 and ShAb02 to RBD molecules
Source data are provided as a Source Data file.

	ShAb01			ShAb02		
	K_D (nM)	k_{on} $(M*s)^{-1}$	k_{off} $(s)^{-1}$	K_D (nM)	k_{on} $(M*s)^{-1}$	k_{off} $(s)^{-1}$
WA-1	47.1 ± 0.713	4.16×10^{4}	1.96×10^{-3}	85.7 ± 2.29	8.12×10^{4}	6.96×10^{-3}
K417N	55.0 ± 0.299	4.51×10^{4}	2.48×10^{-3}	53.9 ± 0.818	1.51×10^{5}	8.15×10^{-3}
E484K, N501Y	73.0 ± 39.6	3.47×10^{4}	2.53×10^{-3}	49.0 ± 0.865	1.14×10^{5}	5.57×10^{-3}
B.1.427/429, Epsilon (L452R)	35.1 ± 0.585	5.74×10^{4}	2.01×10^{-3}	29.8 ± 0.574	1.31×10^{5}	3.91×10^{-3}
B.1.1.7, Alpha (N501Y)	53.9 ± 0.408	4.12×10^{4}	2.22×10^{-3}	72.4 ± 1.03	1.16×10^{5}	8.36×10^{-3}
B.1.351, Beta (K417N, E484K, N501Y)	70.8 ± 0.392	3.73×10^{4}	2.61×10^{-3}	71.6 ± 1.25	9.68×10^{4}	6.93×10^{-3}
B.1.617.2, Delta (L452R, T478K)	38.9 ± 0.303	6.28×10^{4}	2.45×10^{-3}	14.9 ± 0.592	1.88×10^{5}	2.80×10^{-3}
P.1, Gamma (K417T, E484K, N501Y)	86.3 ± 0.661	3.11×10^{4}	2.68×10^{-3}	90.4 ± 2.26	6.50×10^{4}	5.88×10^{-3}
B.1.525, Eta, Iota (E484K)	70.0 ± 0.466	3.73×10^{4}	2.61×10^{-3}	49.6 ± 0.730	1.31×10^{5}	6.50×10^{-3}
B.1.617, Kappa (L452R, E484Q)	31.4 ± 0.385	8.63×10^{4}	2.71×10^{-3}	29.3 ± 0.712	1.68×10^{5}	4.93×10^{-3}
B.1.1.529, Omicron BA.1 (G339D, S371L, S373P, S375F, K417N, N440K, G446S, S477N, T478K, E484A, Q493K, G496S, Q498R, N501Y, Y505H)	7463.1 ± 2230	5.25×10^{3}	3.92×10^{-2}	108.2 ± 2.98	9.16×10^{4}	9.86×10^{-2}
B.1.1.529.2, Omicron BA.2 (G339D, S371L, S373P, S375F, K417N, N440K, G446S, S477N, T478K, E484A, Q493K, G496S, Q498R, N501Y, Y505H)	161.0 ± 4.14	5.99×10^{4}	9.64×10^{-3}	125 ± 5.69	5.30×10^{4}	6.60×10^{-3}
B.1.1.529.2.12.1, Omicron BA.2.12.1 (G339D, S371F, S373P, S375F, T376A, D405N, R408S, K417N, N440K, L452Q, S477N, T478K, E484A, Q493R, Q498R, N501Y, Y505H,)	307.0 ± 12.2	5.83×10^{4}	1.79×10^{-2}	86.5 ± 3.97	7.44×10^{4}	6.43×10^{-3}
Omicron BA.4/5 (G339D, S371F, S373P, S375F, T376A, D405N, R408S, K417N, N440K, G446S, L452R, S477N, T478K, E484A, F486V, G496S, Q498R, N501Y, Y505H)	152.9 ± 5.36	1.02×10^{5}	1.56×10^{-2}	50.1 ± 1.41	9.63×10^{4}	4.82×10^{-3}
SARS-CoV-1 Urbani	198.1 ± 2.81	3.77×10^{4}	7.47×10^{-3}	n/d	n/d	n/d

n/d: not determined

Supplementary Table 3. Crystallography data collection and refinement statistics

	SARS-CoV-2 RBD + ShAb01 VNAR + ShAb02 VNAR
Crystallization condition	14.5% PEG20000, 0.1M HEPES pH7.0
Data collection	
Space group	$P2_12_12_1$
Cell dimensions	
a, b, c (Å)	51.1, 62.8, 147.9
α, β, γ (°)	90, 90, 90
Resolution (Å)	57.8-2.5 (2.6-2.5)
R_{sym}	0.303 (1.598)
I / σI	5.5 (0.94)
Reflections (total/unique)	216,995/ 16,633
Completeness (%)	99.49 (95.51)
Redundancy	13.0 (13.2)
$CC_{(1/2)}$	0.996 (0.375)
R_{pim}	0.0808 (0.8802)
Refinement	
Resolution (Å)	57.8-2.5 (2.6-2.5)
No. reflections	16,573 (1,554)
R_{work} / R_{free} *	23.1/27.9
Ramachandran favored/allowed/outliers	95.7 / 4.1 / 0.2
B-Factor	
Protein/water	82.3/58.8
R.m.s deviations	
Bond lengths (Å)	0.002
Bond angles (°)	0.579
PDB ID	7S83

Supplementary Table 4. ShAb01 interface with RBD

	ShAb01a	RBD	Distance (Å)
HYDROGEN BONDS	A:ALA 1[O]	C:LYS 378[NZ]	2.90
	A:LEU 99[O]	C:SER 383[OG]	2.31
	A:GLY 100[O]	C:TYR 369[OH]	2.68
	A:SER 101[OG]	C:CYS 379[N]	2.81
	A:TYR 102[O]	C:PHE 377[N]	3.13
	A:GLY 98[N]	C:CYS 379[O]	2.51
	A:SER 101[OG]	C:CYS 379[O]	3.51
	A:TYR 102[N]	C:PHE 377[O]	3.20
	A:TYR 102[OH]	C:TYR 369[O]	3.07
	A:TYR 102[OH]	C:SER 371[O]	3.57
	A:TYR 104[N]	C:SER 375[O]	2.97
SALT BRIDGE	A:GLU 103[OE1]	C:LYS 378[NZ]	2.99

Supplementary Table 5. ShAb02 interface with RBD

	ShAb02a	RBD	Distance (Å)
HYDROGEN BONDS	B:GLU 90[N]	C:ARG 355[O]	3.67
	B:LYS 51[N]	C:ASN 450[OD1]	2.80
	B:SER 61[N]	C:THR 470[OG1]	3.10
	B:SER 61[OG]	C:THR 470[OG1]	3.57
	B:ASP 99[OD1]	C:ARG 346[NH1]	2.94
	B:TYR 37[OH]	C:ARG 346[NH2]	2.34
	B:TYR 101[OH]	C:ARG 346[NH2]	3.71
	B:ASP 87[OD1]	C:ASN 354[ND2]	3.65
	B:SER 88[O]	C:ASN 354[ND2]	3.81
	B:SER 88[O]	C:ARG 355[N]	2.66
	B:GLU 90[OE1]	C:ARG 357[NE]	2.90
	B:GLU 90[OE2]	C:TYR 396[OH]	3.70
	B:ILE 49[O]	C:ASN 450[ND2]	3.21
	B:ASP 87[OD1]	C:ARG 466[NH1]	3.03
	B:ASP 87[OD2]	C:ARG 466[NH2]	3.13
	B:SER 61[OG]	C:THR 470[N]	3.09
	B:VAL 59[O]	C:THR 470[OG1]	2.82
SALT BRIDGE	B:ASP 99[OD1]	C:ARG 346[NH1]	2.94
	B:GLU 90[OE1]	C:ARG 357[NE]	2.90
	B:ASP 87[OD2]	C:ARG 466[NH1]	3.90
	B:ASP 87[OD1]	C:ARG 466[NH1]	3.03
	B:ASP 87[OD2]	C:ARG 466[NH2]	3.13
	B:ASP 87[OD1]	C:ARG 466[NH2]	3.63

Supplementary Table 6. Buried surface Area (BSA in Å^2) of ShAb01-RBD

ShAb01	BOND TYPE	BSA
A:ALA 1	H	57.94
A:ARG 2		49.47
A:ARG 8		34.89
A:GLU 46		13.42
A:ARG 82		21.77
A:LYS 84		6.98
A:TYR 86		8.65
A:TRP 87		9.26
A:LYS 94		56.43
A:PHE 95		99.50
A:CYS 96		11.50
A:PRO 97		40.89
A:GLY 98	H	32.18
A:LEU 99	H	56.41
A:GLY 100	H	30.01
A:SER 101	H	46.05
A:TYR 102	H	123.28
A:GLU 103	S	35.43
A:TYR 104	H	56.78
A:ASP 106		27.30

H: HYDROGEN BOND, S: SALT BRIDGE

RBD	BOND TYPE	BSA
C:TYR 369	H	59.70
C:SER 371	H	17.89
C:ALA 372		45.55
C:PHE 374		25.75
C:SER 375	H	54.01
C:THR 376		18.27
C:PHE 377	H	40.84
C:LYS 378	HS	101.24
C:CYS 379	H	24.81
C:TYR 380		57.16
C:GLY 381		6.06
C:VAL 382		5.17
C:SER 383	H	31.24
C:PRO 384		23.69
C:THR 385		20.22
C:LYS 386		4.35
C:ASP 405		0.16
C:ARG 408		78.80
C:ALA 411		3.00
C:PRO 412		28.51
C:GLY 413		19.10
C:GLN 414		13.48
C:ASP 427		39.68
C:ASP 428		20.09
C:PHE 429		3.32
C:GLY 502		3.25
C:VAL 503		64.74
C:TYR 508		2.31

H: HYDROGEN BOND, S: SALT BRIDGE

Supplementary Table 7. Buried surface Area (BSA in Å^2) of ShAb02-RBD

ShAb02	BOND TYPE	BSA
B:CYS 29		0.83
B:ALA 30		22.72
B:LEU 31		11.08
B:ALA 32		49.27
B:SER 33		19.18
B:ASP 35		12.50
B:TYR 37	H	17.97
B:SER 48		5.03
B:ILE 49	H	5.40
B:SER 50		51.02
B:LYS 51	H	63.08
B:GLY 52		4.50
B:GLU 57		2.58
B:THR 58		7.71
B:VAL 59	H	48.38
B:ASN 60		4.19
B:SER 61	H	57.84
B:GLY 62		2.90
B:ASN 84		2.56
B:TRP 86		86.90
B:ASP 87	HS	22.80
B:SER 88	H	55.16
B:TRP 89		100.62
B:GLU 90	HS	95.15
B:THR 91		7.02
B:ARG 92		1.24
B:CYS 96		0.16
B:ASP 97		20.70
B:ASP 99	HS	11.66
B:TYR 101	H	6.92
H: HYDROGEN BOND, S: SALT BRIDGE		

RBD	BOND TYPE	BSA
C:ARG 346	HS	130.75
C:PHE 347		6.65
C:ALA 348		18.24
C:SER 349		1.30
C:TYR 351		18.81
C:ALA 352		20.45
C:TRP 353		8.38
C:ASN 354	H	53.78
C:ARG 355	H	69.81
C:LYS 356		36.80
C:ARG 357	HS	30.20
C:TYR 396	H	20.98
C:LYS 444		25.74
C:GLY 447		3.30
C:ASN 448		1.42
C:TYR 449		26.31
C:ASN 450	H	91.28
C:LEU 452		6.69
C:PHE 464		5.28
C:GLU 465		0.33
C:ARG 466	HS	75.71
C:ILE 468		68.27
C:SER 469		11.18
C:THR 470	H	64.23
C:GLU 471		5.71
C:PHE 490		21.28
C:LEU 492		0.34
H: HYDROGEN BOND, S: SALT BRIDGE		

Supplementary Table 8. Binding kinetics table for multi-specific ShAb molecules to CoV RBDs
Source data are provided as a Source Data file.

	ShAb01H02K			BiShAb0201		
	K_D (nM)	k_{on} $(M*s)^{-1}$	k_{off} $(s)^{-1}$	K_D (nM)	k_{on} $(M*s)^{-1}$	k_{off} $(s)^{-1}$
WA-1	0.57 ± 0.007	1.22×10^{5}	6.98×10^{-5}	1.07 ± 0.026	2.63×10^{4}	2.82×10^{-5}
K417N	1.3 ± 0.065	3.53×10^{5}	4.57×10^{-4}	1.7 ± 0.358	2.11×10^{5}	3.52×10^{-4}
E484K, N501Y	1.9 ± 0.107	2.77×10^{5}	5.12×10^{-4}	2.3 ± 0.415	1.77×10^{5}	4.12×10^{-4}
B.1.427/429, Epsilon (L452R)	0.08 ± 0.431	1.56×10^{5}	1.21×10^{-5}	1.2 ± 0.532	1.09×10^{5}	1.33×10^{-4}
B.1.1.7, Alpha (N501Y)	1.5 ± 0.091	3.41×10^{5}	5.13×10^{-4}	1.8 ± 0.331	2.07×10^{5}	3.70×10^{-4}
B.1.351, Beta (K417N, E484K, N501Y)	1.8 ± 0.083	2.94×10^{5}	5.38×10^{-4}	2.1 ± 0.380	2.06×10^{5}	4.36×10^{-4}
B.1.617.2, Delta (L452R, T478K)	<0.001 ± 0.004	9.00×10^{4}	$<1 \times 10^{-7}$	0.72 ± 0.008	5.7×10^{4}	4.10×10^{-5}
P.1, Gamma (K417T, E484K, N501Y)	4.2 ± 0.806	7.35×10^{4}	3.05×10^{-4}	6.5 ± 0.752	7.80×10^{4}	5.08×10^{-4}
B.1.525, Eta, Iota (E484K)	1.6 ± 0.098	3.10×10^{5}	5.11×10^{-4}	2.0 ± 0.255	2.10×10^{5}	4.15×10^{-4}
B.1.617, Kappa (L452R, E484Q)	2.2 ± 0.430	1.51×10^{5}	3.26×10^{-4}	4.4 ± 0.328	5.36×10^{4}	2.38×10^{-4}
B.1.1.529, Omicron BA.1 (G339D, S371L, S373P, S375F, K417N, N440K, G446S, S477N, T478K, E484A, Q493K, G496S, Q498R, N501Y, Y505H)	24.5 ± 24.5	7.13×10^{4}	1.75×10^{-3}	15.1 ± 1.47	1.02×10^{5}	1.54×10^{-3}
B.1.1.529.2, Omicron BA.2 (G339D, S371L, S373P, S375F, K417N, N440K, G446S, S477N, T478K, E484A, Q493K, G496S, Q498R, N501Y, Y505H)	24.1 ± 1.63	7.70×10^{4}	1.86×10^{-3}	33.2 ± 1.89	2.26×10^{4}	7.49×10^{-4}
B.1.1.529.2.12.1, Omicron BA.2.12.1 (G339D, S371F, S373P, S375F, T376A, D405N, R408S, K417N, N440K, L452Q, S477N, T478K, E484A, Q493R, Q498R, N501Y, Y505H)	17.4 ± 1.24	9.85×10^{4}	1.71×10^{-3}	46.9 ± 2.70	1.97×10^{4}	9.22×10^{-4}
Omicron BA.4/5 (G339D, S371F, S373P, S375F, T376A, D405N, R408S, K417N, N440K, G446S, L452R, S477N, T478K, E484A, F486V, G496S, Q498R, N501Y, Y505H)	5.6 ± 0.43	1.23×10^{5}	6.92×10^{-4}	14.9 ± 0.91	5.15×10^{4}	7.67×10^{-4}
SARS-CoV-1 Urbani	122.9 ± 1.10	6.95×10^{4}	8.54×10^{-3}	102.3 ± 0.971	1.03×10^{5}	1.06×10^{-2}
