# Supplementary material for: Shark nanobodies with potent SARS-CoV-2 neutralizing activity and broad sarbecovirus reactivity
Source: Nat Commun. 2023 Feb 3;14:580. doi: 10.1038/s41467-023-36106-x (PMC9896449; doi:10.1038/s41467-023-36106-x)
Supplement: Supplementary file 2 — Reporting Summary [file 41467_2023_36106_MOESM2_ESM.pdf]

nature portfolio

Corresponding author(s): MG Joyce, H Dooley

Last updated by author(s): 12/20/2022

Reporting Summary

Nature Portfolio wishes to improve the reproducibility of the work that we publish. This form provides structure for consistency and transparency in reporting. For further information on Nature Portfolio policies, see our Editorial Policies and the Editorial Policy Checklist.

Statistics

For all statistical analyses, confirm that the following items are present in the figure legend, table legend, main text, or Methods section.

n/a	Confirmed	
[ ]	[x]	The exact sample size (n) for each experimental group/condition, given as a discrete number and unit of measurement
[ ]	[x]	A statement on whether measurements were taken from distinct samples or whether the same sample was measured repeatedly
[ ]	[x]	The statistical test(s) used AND whether they are one- or two-sided<br>*Only common tests should be described solely by name; describe more complex techniques in the Methods section.*
[x]	[ ]	A description of all covariates tested
[ ]	[x]	A description of any assumptions or corrections, such as tests of normality and adjustment for multiple comparisons
[ ]	[x]	A full description of the statistical parameters including central tendency (e.g. means) or other basic estimates (e.g. regression coefficient) AND variation (e.g. standard deviation) or associated estimates of uncertainty (e.g. confidence intervals)
[ ]	[x]	For null hypothesis testing, the test statistic (e.g. F, t, r) with confidence intervals, effect sizes, degrees of freedom and P value noted<br>*Give P values as exact values whenever suitable.*
[x]	[ ]	For Bayesian analysis, information on the choice of priors and Markov chain Monte Carlo settings
[x]	[ ]	For hierarchical and complex designs, identification of the appropriate level for tests and full reporting of outcomes
[x]	[ ]	Estimates of effect sizes (e.g. Cohen's d, Pearson's r), indicating how they were calculated

Our web collection on statistics for biologists contains articles on many of the points above.

Software and code

Policy information about availability of computer code

Data collection	Diffraction data were collected at APS 24-ID-E beamline using RAPD, a modular package of programs written for the automated processing of macromolecular crystallographic data. Final Diffraction data indexing, integration, and scaling were carried out using the HKL2000 suite.
Data analysis	Visual analysis and figure generation was conducted using ChimeraX 1.1 and PyMOL 2.3.2. Affinity kinetic constants between SARS-CoV-2 RBD molecules and ShAbs were determined using Data analysis software 9.0 (ForteBio). Phenix.xtriage was used to analyze all the scaled diffraction data output from HKL2000 and XDS. Primarily, data was analyzed for measurement value significance, completeness, asymmetric unit volume, and possible twinning and/or pseudotranslational pathologies. The crystal structure described in this study was solved by molecular replacement using the program Phaser. Refinement for the structure model was carried out using Phenix refine with positional, global isotropic B-factor refinement and defined TLS groups. Manual model building was performed in Coot 0.8.9.2. All structure figures were generated using PyMOL 2.3.2 (The PyMOL Molecular Graphics System, Schrodinger).

For manuscripts utilizing custom algorithms or software that are central to the research but not yet described in published literature, software must be made available to editors and reviewers. We strongly encourage code deposition in a community repository (e.g. GitHub). See the Nature Portfolio guidelines for submitting code & software for further information.

Data

Policy information about availability of data

All manuscripts must include a data availability statement. This statement should provide the following information, where applicable:

- Accession codes, unique identifiers, or web links for publicly available datasets
- A description of any restrictions on data availability
- For clinical datasets or third party data, please ensure that the statement adheres to our policy

The atomic model is deposited in the PDB under accession number PDB 7S83. All other data are available in the main manuscript or extended data section.

Human research participants

Policy information about studies involving human research participants and Sex and Gender in Research.

Reporting on sex and gender	N/A
Population characteristics	N/A
Recruitment	N/A
Ethics oversight	N/A

Note that full information on the approval of the study protocol must also be provided in the manuscript.

Field-specific reporting

Please select the one below that is the best fit for your research. If you are not sure, read the appropriate sections before making your selection.

☒ Life sciences ☐ Behavioural & social sciences ☐ Ecological, evolutionary & environmental sciences

For a reference copy of the document with all sections, see nature.com/documents/nr-reporting-summary-flat.pdf

Life sciences study design

All studies must disclose on these points even when the disclosure is negative.

Sample size	Sample size for the K18-hACE2 animal groups was n=13/group, 7 female, 6 male. From this initial group, n=8 animals were assessed for 14 days for weight, clinical signs, and mortality. Calculations indicated that a 60% incidence rate, with a power of 80%, group sizes of 8 animals would be adequate. Post-hoc analysis of the results indicates that this group size was powered for the study.<br><br>Shark group sizes were chosen as a minimal sample size to allow identification of antigen-specific shark nanobodies.
Data exclusions	No data exclusions were performed.
Replication	All attempts at replication were successful. Antibody affinity measurements were carried out in duplicate, with at least four concentrations used for each of the RBD molecules. ELISA assays were carried out in duplicate for each assay, with at least one replicate experiment also completed.
Randomization	No randomization of samples was carried out.
Blinding	Investigators carrying out the K18-hACE2 animal challenge studies were blinded to the ShAb identity used in the study. No other blinding was carried out for other experiments described in the manuscript.

Reporting for specific materials, systems and methods

We require information from authors about some types of materials, experimental systems and methods used in many studies. Here, indicate whether each material, system or method listed is relevant to your study. If you are not sure if a list item applies to your research, read the appropriate section before selecting a response.

Materials & experimental systems

n/a	Involved in the study
	x Antibodies
	x Eukaryotic cell lines
x	Palaeontology and archaeology
	x Animals and other organisms
x	Clinical data
x	Dual use research of concern

Methods

n/a	Involved in the study
x	ChIP-seq
x	Flow cytometry
x	MRI-based neuroimaging

Antibodies

Antibodies used	1. Anti-Human IgG (gamma-chain specific)-Peroxidase antibody produced in goat (Sigma, catalogue number A8419-2ML)<br>2. Anti-M13 phage coat G8p mAb (RL-ph1) (Invitrogen, catalogue number MA1-06603).<br>3. Anti-Mouse IgG (whole molecule)–Peroxidase antibody produced in sheep (Sigma, catalogue number A6782).<br>4. Anti-Zika virus antibody (MZ4), supplied by the Krebs Lab (WRAIR).
Validation	1. Specific for human IgG gamma chain. The cross-reactivities of anti-human IgG (gamma) antibody are tested in an ELISA. Minimal cross-reactivity to light chains. The reagent is an affinity purified antibody from goat. The purified antibody is conjugated to horseradish peroxidase (HRP) and stabilized in buffer.<br>2. Source: Recombinant murine IgG2a/K. Immunogen: M13 phage. Specificity: Major coat protein g8p of M13 filamentous bacteriophage. Clone: A4B1. Purification: Protein A affinity chromatography. Purity: >95%.<br>3. Source: sheep. Immunogen: mouse IgG (whole molecule). Specificity: reacts with mouse IgG in vitro and in mouse serum and biological fluids. It does not react with human serum proteins.<br>4. Source: Recombinant human IgG1/K. Immunogen: Zika Purified Inactivated vaccine. Specificity: Zika E glycoprotein. Clone: MZ4. Purification: Protein A affinity chromatography. Purity: >95%.

Eukaryotic cell lines

Policy information about cell lines and Sex and Gender in Research

Cell line source(s)	Expi293F (Thermo Fisher Scientific, A14635), and HEK-293T (ATCC CRL-3216) mammalian cells.
Authentication	The cell lines were not authenticated.
Mycoplasma contamination	The cells were not tested for mycoplasma contamination.
Commonly misidentified lines (See ICLAC register)	No commonly misidentified cell lines were used in this study.

Animals and other research organisms

Policy information about studies involving animals; ARRIVE guidelines recommended for reporting animal research, and Sex and Gender in Research

Laboratory animals	Transgenic mice, B6.Cg-Tg(K18-ACE2)2Prlmn/J, common name: K18-hACE2, male, female, 6-8 weeks of age. Animals were maintained in IVC cages on negatively pressurized Allentown PNC racks that were HEPA filtered and directly vented though the building's exhaust system. The racks and animal rooms were negatively pressurized. Access to all facilities is controlled electronically by the buildings management systems and restricted to approved users only. The environment, temperature, and humidity, within the animal facility room were constantly monitored by the building management system. Temperatures were also monitored and recorded daily in individual animal rooms by animal care staff using electronic thermometers. All temperature set points were within The Guide recommended range of 68-79°F for mice. Acceptable Institutional daily fluctuations are between 67-74°F with a humidity range of 30-70%. Light cycles in all animal holding and procedure spaces are controlled on a 12/12 light/dark cycle.<br><br>Nurse sharks (Ginglymostoma cirratum). Six juvenile nurse sharks (two males and four females, aged between 2-3 years and weighing between 1.8-3.8 kg), were held in a continuously-recirculating 12,000L seawater tank maintained at 28°C, in the Aquaculture Research Center at the Institute of Marine & Environmental Technology (IMET), Baltimore, USA.
Wild animals	The study did not involve wild animals.
Reporting on sex	Six juvenile nurse sharks (two males and four females) were used in the immunization portion of the study. Sex-based analyses were not performed as the study was not designed with sufficient power to differentiate across groups based on sex.<br><br>K18-hACE2 mouse challenge study findings apply to both sexes. Both female and male mice were included in the challenge study. Sex-based analyses were not performed as the study was not designed with sufficient power to differentiate across groups based on sex.<br>ShAb01 group: females 1/4 mice died at day 9, males 0/3 mice died<br>ShAb02 group: females 1/4 mice died at day 7, males 3/3 mice died (2 on day 5, and 1 on day 6)

	Isotype control group: females 5/5 mice died, males 5/5 mice died. All mice died between day 6 and day 8.
Field-collected samples	The study did not involve samples collected from the field.
Ethics oversight	All research in this study involving animals was conducted in compliance with the Animal Welfare Act, and other federal statutes and regulations relating to animals and experiments involving animals and adhered to the principles stated in the Guide for the Care and Use of Laboratory Animals, NRC Publication, 1996 edition.<br>The sharks used in this study were acquired under a Special Activity License granted by the Florida Fish and Wildlife Conservation Commission. All animal procedures were conducted under protocol #0318003 in accordance with University of Maryland, School of Medicine Institutional Animal Care and Use Committee (IACUC)- and USAMRDC Animal Care and Use Review Office (ACURO)-approved protocols.<br>The K18-hACE2 transgenic mice research protocol was approved by the Institutional Animal Care and Use Committee of the Trudeau Institute, protocol 20-007. K18-hACE2 transgenic mice were obtained from Jackson Laboratories (Bar Harbor, ME). Mice were housed in the animal facility of the Trudeau Institute and cared for in accordance with local, state, federal, and institutional policies in a National Institutes of Health American Association for Accreditation of Laboratory Animal Care-accredited facility.

Note that full information on the approval of the study protocol must also be provided in the manuscript.
